# Problems encountered with the use of simulation in an attempt to enhance interpretation of a secondary data source in epidemiologic mental health research

**DOI:** 10.1186/1756-0500-3-231

**Published:** 2010-08-26

**Authors:** Scott B Patten

**Affiliations:** 1 Department of Community Health Sciences, University of Calgary, 4thFloor TRW Building, 3280 Hospital Drive N.W., Calgary, Alberta, T2N 4N1, Canada; 2 Hotchkiss Brain Institute, Health Research & Innovation Centre, Room 1A10, 3330 Hospital Dr. N.W. Calgary, Alberta, T2N 4N1, Canada

## Abstract

**Background:**

The longitudinal epidemiology of major depressive episodes (MDE) is poorly characterized in most countries. Some potentially relevant data sources may be underutilized because they are not conducive to estimating the most salient epidemiologic parameters. An available data source in Canada provides estimates that are potentially valuable, but that are difficult to apply in clinical or public health practice. For example, weeks depressed in the past year is assessed in this data source whereas episode duration would be of more interest. The goal of this project was to derive, using simulation, more readily interpretable parameter values from the available data.

**Findings:**

The data source was a Canadian longitudinal study called the National Population Health Survey (NPHS). A simulation model representing the course of depressive episodes was used to reshape estimates deriving from binary and ordinal logistic models (fit to the NPHS data) into equations more capable of informing clinical and public health decisions. Discrete event simulation was used for this purpose. Whereas the intention was to clarify a complex epidemiology, the models themselves needed to become excessively complex in order to provide an accurate description of the data.

**Conclusions:**

Simulation methods are useful in circumstances where a representation of a real-world system has practical value. In this particular scenario, the usefulness of simulation was limited both by problems with the data source and by inherent complexity of the underlying epidemiology.

## Findings

Major Depressive Disorder (MDD) is a mood disorder that is characterized by one or more major depressive episodes (MDE). Clinical practice guidelines for MDD have historically regarded the diagnosis as a *de facto *indication of treatment need (e.g.[1]). However, in community studies application of diagnostic criteria for MDD has been shown to identify some short lived episodes that may not be associated with a need for treatment [2]. This has led to more recent recommendations acknowledging the apparent heterogeneity of this condition. For example, in the strategy of "watchful waiting" treatment may be delayed for several weeks while there is ongoing monitoring in order to determine whether an episode will resolve without active treatment [3]. For mild episodes, guided self-management has also been proposed as a reasonable intervention [3,4].

It would be helpful to make use of epidemiologic data in order to quantify the probability of various outcomes and ultimately to use this information as a means of supporting clinical decisions. Recently, the predictD study has reported predictive algorithms for the risk of MDE in general practice attendees in six European countries [5]. The algorithm was subsequently externally validated in Chilean general practices and has been made available via the internet [6]. The predictD group reported that many factors predicting incidence of MDE also predict persistence of symptoms, albeit with generally weaker associations being observed in predictive models [7]. Another project leading to the development of an algorithm for predicting prognosis of MDE in primary care was reported by Rubinstein et al. [8]. No such studies have been conducted in general population samples.

Epidemiologic data are useful for understanding the determinants of MDD, but in many countries existing data sources have limitations. In Canada, for example, there is a longitudinal data source arising from a general population sample called the National Population Health Survey (NPHS) that can potentially assist with these objectives. This study has followed a representative cohort of community (household) residents since 1994. However, direct estimates made from the NPHS do not always align well with the research needs identified above because of limitations and qualifications of the data. For example, the NPHS measures past-year MDE during interviews conducted two years apart, an approach that does not precisely align with the concept of risk. In addition, the NPHS measures weeks depressed during the preceding year, which is not the same as episode duration - the latter being a more salient parameter for practice. These problems are not unique to Canada. Many longitudinal studies employ depression measures that share analogous limitations. Some longitudinal studies, for example, obtain symptom ratings using scales that cover past week symptoms, but these ratings are spaced apart more widely (e.g. administered annually).

In the current project, a simulation model was developed in order to represent relevant probabilities in ways that align more closely with clinical needs than would direct estimates from a flawed epidemiologic data source such as the NPHS. The simulation model provided a representation of the epidemiology over a four year period. Inputs to the model were parameters representing easily interpretable epidemiologic estimates and the model was programmed to produce outputs representing the various flawed estimates that can be directly estimated from the NPHS data. Simulations using various values for the input parameters were then used to identify values that would be expected to reproduce the less interpretable ones that can be directly estimated. In other words, estimates from the NPHS were used to calibrate the simulation model and the simulation model was taken as a representation of the underlying epidemiology. The objective was to produce a set of easily interpretable estimates of conventional parameters such as odds ratios and hazard ratios from the less conventional and less easily interpretable estimates that can be directly estimated from the NPHS.

The NPHS is a longitudinal study of a nationally representative community sample assembled by Statistics Canada in 1994/1995. NPHS respondents are interviewed every two years, with data currently being available up to 2006. Detailed information about NPHS methodology is available on the Statistics Canada Web page [9]. The sampling frame was restricted to residents of private dwellings and excluded people residing in institutions, First Nation Reserves, members of the Armed Forces and certain remote locations. The longitudinal cohort initially included 17,276 participants, but the current analysis was restricted to 15,254 respondents who were 12 years or older at the time of the initial 1994/1995 interview. Over the subsequent 12 years (the period for which data are currently available), 13.2% of the original cohort was lost to follow-up due to refusal and 5.4% were lost to follow-up due to failure to track. By 2006, 3,977 members of the longitudinal cohort were classified as non-respondents, leading to an estimated response rate of 77.0%. Another 2,032 were deceased and 148 were institutionalized prior to the 2006 interview. These participants are not treated as non-responders in the response rate calculation since they were followed successfully until they left the sampling frame [10]. Most (75%) of the 1994 baseline interviews were conducted in person, but almost all (99%) of follow-up interviews were conducted over the phone.

The NPHS included a diagnostic instrument called the Composite International Diagnostic Interview short form for major depression (CIDI-SFMD)[11]. This brief fully-structured diagnostic interview was administered by experienced and trained lay interviewers working with Statistics Canada. The CIDI-SFMD is scored with a predictive probability algorithm that incorporates the number of symptom-based criteria fulfilled during a 2-week period in the preceding year. The scoring algorithm was developed using a receiver-operator analysis relating short form scores to DSM-IIIR diagnoses obtained from a full-length version of the CIDI in the US National Comorbidity Survey [11]. The algorithm requires either depressed mood or loss of interest or pleasure during the same 2 week period, consistent with DSM-IV criteria [12]. The CIDI-SFMD was scored using an algorithm expected to produce a 90% positive predictive value. This scoring algorithm requires endorsement of 5 depressive symptom-based criteria during the same 2-week period, consistent with DSM-IV criteria. Respondents with past-year MDE were also asked to report the number of weeks in which they were depressed in the year preceding the interview.

The NPHS interview also measures a set of variables that may be useful in predicting the course of depression. The association of a set of variables (listed below) with aspects of MDD prognosis was evaluated, but only variables found to be significantly associated with outcome (see below) were included in the simulation models. In the NPHS, standard items are used to assess age, sex, marital status, income adequacy (total household income adjusted for family size), employment status, the presence of one or more chronic medical conditions, a history of several childhood stressors (spending two weeks for more in hospital, parental divorce, parental unemployment, being sent away from home for doing something wrong, problematic parental drug or alcohol use, physical abuse by someone close), pain (pain was categorized using two items from the Health Utility Index [13]: (i) Are you usually free of pain or discomfort? (ii) How would you describe the usual intensity of your pain or discomfort? Among those reporting that they were not usually free of pain or discomfort, those who characterized their pain as "severe" in response to the second item were coded positively), family history of depression (professionally diagnosed depression among first degree relatives), self-esteem & mastery [14], excessive alcohol consumption (here defined as > 5 standard drinks on any one occasion) and current smoking status were also assessed. Distress was measured using the K-6 scale [15]. Age was categorized into the following groups for purposes of analysis: 12-18 years, 19 to 25 years, 26 to 45 years, 46 to 65 years and 66 of more years.

For those respondents identified as having a depressive episode according to the CIDI-SFMD an additional item determined the number of weeks in the preceding year that they were depressed. In analysis, responses from all of the seven available NPHS cycles were combined into a single data file in order to derive more precise estimates of the proportions reporting various numbers of weeks depressed. In order to ensure independence of the observations, only a single episode from each respondent (the first episode) was included in situations where multiple episodes occurred during follow-up. Also, only apparently new episodes were included (those without a positive CIDI-SFMD rating in the preceding cycle) in order to minimize the possibility that a long episode from a previous cycle might resolve during the follow-up interval and be misconstrued as a brief episode. The combination of data for weeks depressed in past year from various cycles into a single data file resulted in estimates of weeks depressed in past year for a total of n = 1477 episodes in the same number of NPHS respondents.

The weeks depressed in past year variable is not the only prognostic information available in the NPHS. As the NPHS follows a cohort prospectively, it was also possible to identify the proportion of respondents with a new episode at any cycle who were again positive on the CIDI-SFMD at the subsequent cycle 2 years later. All respondents with new onset (prior cycle was CIDI-SFMD negative) episodes were identified and the proportion of these that were associated with another positive CIDI-SFMD result 2 years later was estimated. Again, to ensure independence of observations, only one episode for an individual respondent was included in this data file. The data file contained n = 1857 respondents.

After preliminary tabular and stratified analyses, several statistical approaches were used to model the relationship of potential determinants to the longitudinal course of the depressive episodes. Associations between weeks depressed in past year categories and various potential determinants were modelled using ordinal logistic regression. Brant's test was used to assess the proportional odds assumption in the ordinal logistic analysis [16]. Binary logistic regression was used to model the proportion of those with episodes who had consecutively positive CIDI-SFMD ratings (persistent or recurrent episodes).

The analysis was conducted at the Prairie Regional Research Data Centre on the University of Calgary campus, using STATA [17]. The study received approval from the University of Calgary Conjoint Ethics Board.

The goal of simulation in this study was to realign the NPHS data into a format that would more directly relate to clinical and public health practice, see Table 1. The strategy of simulation, as described above, involved producing a representation of the underlying epidemiology and using NPHS estimates as a calibration standard for this representation. In other words, the epidemiology is represented in the simulation model using equations that support prediction of the clinical course, but that can also be used to calculate the flawed parameters that can actually be measured and modelled in the NPHS (weeks depressed in past year, probability of two consecutive CIDI-SFMD past-year episodes). As such, the equations predicting clinical course are to a large extent constrained by the observed values.

**Table 1:** Available data in the NPHS, and associated measures of greater salience to practice

Concept	Available NPHS measure	More salient measure
Episode duration	Weeks depressed in past year	Episode duration*

Recurrence pattern	Episodes present during initial and subsequent cycles	Recurrence risk or rate**

* for example, mean or median episode duration, or the proportion of episodes having a duration falling within a specific range of weeks depressed

** for example, the proportion recovering from an episode who experience a recurrence within a specified interval of time.

The simulation model was developed using discrete event simulation in the software Arena [18]. The model sequentially simulated the experience of "entities" representing people. These entities were assigned attributes representing specific personal characteristics that may predict course, see above. In keeping with the use of terminology in discrete event simulation, these characteristics are subsequently referred to as attributes. The experience of each entity was simulated for two years (not including a two year run-in period), in keeping with the two year interval between NPHS interviews. Since all respondents included in the analysis had a new onset MDE, each entity was assigned a date of onset randomly during the 104 weeks preceding the simulated baseline interview. This strategy was intended to represent the occurrence of new episodes after an initial interview had not detected an episode. The attributes included in the model were selected based on a conventional analysis of the NPHS data that identified characteristics predictive of weeks depressed in past year and the probability of having a MDE again two years later at the subsequent cycle. In an ordinal logistic regression model predicting several categories of weeks depressed in the past year age was the only significant predictor, whereas in binary logistic regression models predicting positive MDE status at a subsequent cycle four predictive variables were identified: age 26-45, smoking, reporting childhood stressors and pain. The simulation model therefore depicted 40 (5 × 2 × 2 × 2) different types of entities having various combinations of these characteristics.

**Figure 1:**
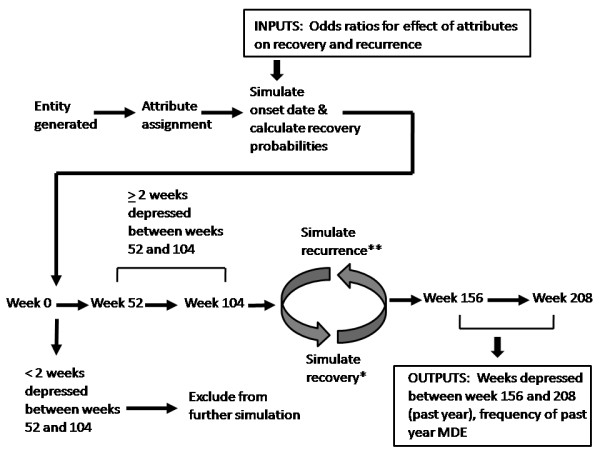
**Schematic depiction of the discrete event simulation model**. * see Equations 1 & 2. ** see Equation 3 and the more elaborate equation in Table 5

The simulation model is depicted graphically in Figure 1. Recovery from an episode was simulated by calculating a recovery probability for each attribute combination during each week of an episode, until recovery occurred or the end-point of the simulation (208 weeks) was reached. The weekly probability of recovery was represented using a transformed linear equation that included log time (in weeks) along with parameters representing the entity's attribute status, see Equation 1. This is consistent with previous simulation work indicating that the probability of recovery can be represented using a Weibull distribution [19,20].

(1) $$\begin{array}{l} {\text{linear predictor}}_{\text{recovery}}=\alpha +\beta_{\text{age}19\text{to}25}*{\text{X}}_{\text{age}19\text{to}25}+\beta_{\text{age26to4}5}*{\text{X}}_{\text{age}26\text{to4}5}+\\ \beta_{\text{age46to6}5}*{\text{X}}_{\text{age46to6}5}+\beta_{\text{age66}\;\text{or more}}*{\text{X}}_{\text{age66}\;\text{to}\;\text{more}}+\beta_{\log\;\text{time}}*\text{Log}\;\text{time}\;\text{(weeks)} \end{array}$$

In Equation 1 the 'X' variables are dummy variables representing the age-grouping attributes assigned to each entity. While depressed, each entity completed a loop in the discrete event simulation modelling path and with each completion of this loop a counter variable representing weeks depressed was programmed to increase by one week. This allowed the probability of recovery in a subsequent week to be adjusted with each passing week's increase in episode duration. Consistent with the wording of the NPHS item, this counter did not distinguish between weeks depressed and consecutive weeks depressed in a single episode. Entities could recover from MDE and have a recurrence during the simulation. In each case there weeks depressed were counted by the model. The linear predictor was transformed into a probability of recovery by the equation [21]:

(2) $$\text{Recovery probability}\;\text{=}\;{\text{1-(exp(-(exp(linear predictor}}_{\text{recovery}}))))$$

Upon recovery, an entity was subject to a weekly risk of recurrence that depended on its attribute status. In the simulation, the weekly probability of recurrence was also calculated based on the entity's attributes using an approach akin to logistic regression. A linear equation was programmed into the model to calculate a value for each attribute found to be associated with MDE recurrence:

(3) $$\begin{array}{l} {\text{linear predictor}}_{\text{weekly recurrence}}=\alpha +\beta_{\text{age}19\text{to}25}*{\text{X}}_{\text{age}19\text{to}25}+\beta_{\text{age26to4}5}*{\text{X}}_{\text{age}26\text{to4}5}+\\ \beta_{\text{age46to6}5}*{\text{X}}_{\text{age46to6}5}+\beta_{\text{age66+}}*{\text{X}}_{\text{age66+}}+\beta_{\text{pain}}*X_{\text{pain}}+\beta_{\text{smoker}}\text{*}\;{\text{X}}_{\text{smoker}}\text{+}\\ \beta_{\text{childhood stressor}}{\text{*X}}_{\text{childhood stressor}} \end{array}$$

The other variables listed in the Methods section were not found to be significantly associated with consecutive episodes in the NPHS analysis. The value returned by this equation was conceptualized as a weekly log odds of recurrence and this was converted into a probability by taking the inverse log and converting the resulting odds to a probability by dividing it by 1 + the odds. In this way, the model calculated a weekly recurrence risk for each recovered entity, depending on that entity's various attribute values. During calibration of the model it was found that this equation was inadequate as a representation of the recurrence rate. This resulted from the effect of age, but not other variables, on weeks depressed in the past year according the ordinal logistic analysis, see above. Since episodes in older age groups were longer than those in younger age groups, a greater proportion of consecutive MD across cycles was attributable to persistence as opposed to recurrence in the older age categories. An expanded version of this equation was therefore devised that included cross-product terms so that the effect of predictive variables on recurrence could vary across age groups. As there was no clear way to determine the minimum necessary set of such cross-product terms, a comprehensive set of such terms was added to the Equation during model calibration.

In order to select appropriate parameters for the α and β coefficients in the equations above, the ordinal and binary logistic models presented in Table 2 and Table 3 (see below) were used. These models were fit directly to the NPHS data. In each case, a fitted value from the model was calculated for each of the forty unique groups defined by the various attribute combinations. A set of coefficients were then explored to determine which combinations of values provided output most closely resembling these models. The software Arena [18] provides a utility (called OptQuest) that can automate this process. The simulation model was programmed to calculate variables representing the sum of squared differences between NPHS values (as predicted from the equations depicted in Table 2 and Table 3, below) and the analogous estimates derived by simulation. OptQuest was then given the task of running multiple simulations using various values for the coefficients listed in Equations 1 and 3, seeking those equations that minimized this sum of squares value.

**Table 2:** Ordinal logistic model for weeks depressed in the past year, in four categories*

Age Group	Coefficient	Odds Ratio	95% Confidence Interval	p-value**
Age 19-25	-.058	0.9	0.6 - 1.5	0.814

Age 26-45	.166	1.2	0.8 - 1.8	0.439

Age 46-65	.441	1.6	1.0 - 2.4	0.047

Age 66 or more	.486	1.6	1.0 - 2.8	0.075

* the 12-18 age group is a baseline category in the model.

** overall likelihood ratio test (chi-square = 13.13, d.f. = 4, p = 0.01)

**Table 3:** Logistic regression model for second CIDI-SF positive interview two years after an initial positive one

	OR	**95% C.I**.	p-value
Age 26-45	1.5	1.0 - 2.1	0.033

Smoker	1.5	1.1 - 2.1	0.024

Childhood Stressor	2.0	1.4 - 2.8	< 0.001

Pain	1.6	1.0 - 2.3	0.033

As noted, in the ordinal logistic regression analysis, a significant association between age group and reported weeks depressed in the past year was observed. This model is presented in Table 2. In this analysis, the Brant test of the parallel regression assumption was non-significant (chi-squared 12.9, d.f. = 12, p = 0.38). None of the other variables listed above were found to be associated with weeks depressed in past year, including sex (OR = 1.1, 95% CI 0.8 - 1.5).

In the logistic regression analysis, age 26 to 45 years was also found to be associated with the probability of having past year MDE two years after an initial MDE, see Table 3. However, three additional variables were also associated: pain, smoking and reporting childhood stressors. See Table 3 for details of the logistic regression model.

The optimized values for the alpha and beta coefficients listed in Equations 1 and 3 are presented in Table 4 and Table 5. As noted above, it was necessary to introduce cross-product terms for the various parameters listed in Equation 3 representing age group by exposure variable interactions.

**Table 4:** Predictive equation for episode duration, from the simulation model

Coefficient*	Estimated value (from simulation)
α	-1.31

β_age19to25_	-0.02

β_age26to45_	-0.13

β_age46to65_	-0.33

β_age66 or more_	-0.35

β_log time_	-0.53

* symbols are those of Equation 1

**Table 5:** Predictive equation for the weekly recurrence rate

Model parameter	Estimate
Intercept	-6.625

Age 19-25	-0.025

Age 26-45	-0.315

Age 46-65	-0.815

Age 66 or more	-4.0

Pain	0.40

Smoking	0.39

Childhood stressor	0.715

Pain by age 26-45	0.08

Pain by age 46-65	0.375

Pain by age 66 or more	3.02

Smoking by age 26-45	0.05

Smoking by age 46-65	0.35

Smoking by age 66 or more	3.0

Childhood stressor by age 26-45	0.03

Childhood stressor by age 46-65	0.45

Childhood stressor by age 66 or more	3.24

Pain by smoking	0.12

Pain by smoking by age 19-25	0.10

Pain by smoking by age 46-65	-0.20

Pain by smoking by age 66 or more	-2.475

Pain by childhood stressor by age 19-25	0.08

Pain by childhood stressor by age 46-65	-0.14

Pain by childhood stressor by age 66 or more	-2.30

Smoking by childhood stressor by age 46-65	-0.11

Smoking by childhood Stressor by age 66 or more	-2.30

Pain by smoking by childhood stressor	-0.035

Pain by smoking by childhood stressor by age 19-25	-0.14

Pain by smoking by childhood stressor by age 26-45	-0.20

Pain by smoking by childhood stressor by age 46-65	-0.075

Pain by smoking by childhood stressor by age 66 or more	1.705

**Figure 2:**
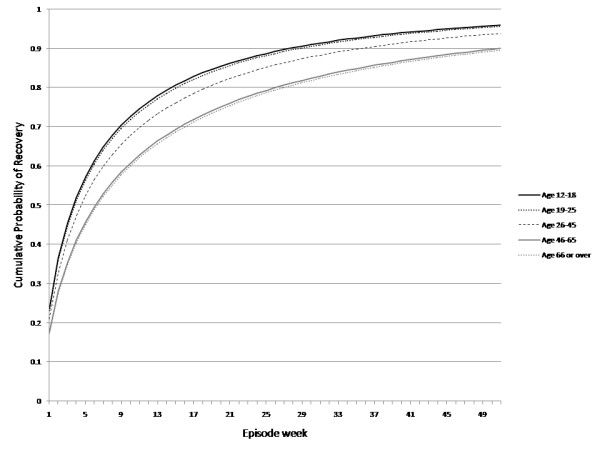
**Simulated cumulative probabilities of recovery**.

Predictions from the model for weeks depressed in past year across the three age groups are depicted in Figure 2. The estimates in Figure 2 are those produced by Equation 2, but expressed as a cumulative function. The simulated frequency of weeks depressed in past year categories are juxtaposed against fitted values from the models fit directly to the NPHS data (see Table 2 and Table 3) and are presented in Additional File 1. In general, the simulation model tended to slightly overestimate the proportion in the 13 to 25 weeks duration category and to slightly underestimate the proportion in the 7 to 12 week category. This appears to have resulted from the way in which the recovery distribution was simulated. In view of the way that the recovery distribution was simulated, the six week period between 7 and 12 weeks tended to have a smaller simulated frequency recovering than the longer (13 week) 13 to 25 week interval. Nevertheless, the simulation provides a reasonable representation of the distribution of episode durations.

**Figure 3:**
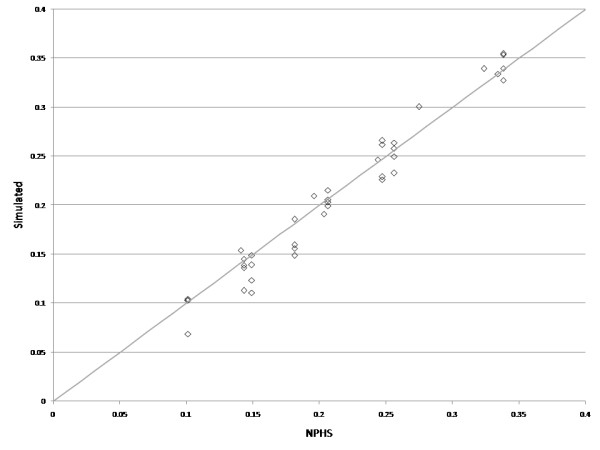
**Scatter plot of fitted versus simulated proportions with MDE in two consecutive cycles**.

Figure 3 shows the simulated frequency of MDE at a subsequent cycle after an episode had occurred at the previous cycle plotted against direct estimates for the 40 modelled groups. The simulation model produced output similar to the direct estimates.

The objective of this study was to increase the utility of longitudinal major depression data collected in a national general health survey. As with some other sources of longitudinal data on depression, the NPHS supports direct estimation of parameters that are, unfortunately, not easily interpretable. The goal was to make use of a representation (in the form of a simulation model) of the underlying epidemiology in order to derive more straightforward estimates from these flawed ones, accounting for the design features of the data source. However, the complexity of the underlying epidemiology combined with aforementioned limitations of the data source precluded full achievement of this objective. One of the accessible proportions in this dataset was the proportion of those depressed at one time interval who were also depressed at a subsequent interval. This proportion represents some mixture of persistent and recurrent cases and a partitioning of these possibilities was one goal of the simulation model, the model also being informed by the observed pattern of recovery. The available information about episode duration (weeks depressed in the past year) indicated that age was an important determinant of this variable whereas a logistic regression model estimating the frequency of positive status on two consecutive cycles was only influenced by age in one age group. If this is true, the effect of age on recurrence must differ depending on exposure to various predictive variables, resulting in the need for very complex equations in the simulation model in order for it to accurately reflect the epidemiology. The equation for recurrence needed to include multiple cross-product terms, see Table 5. Previous studies have identified several variables other than age that may predict episode duration [7]. If the NPHS lacked power to detect some of these associations, the simulated parameters representing both recovery and recurrence would have been affected. Thus, to an unknown extent, the complexity of the simulation may result from actual complexity of the epidemiology or to an artefact of the methodological approach employed. In the end, rather than simply transforming a set of opaque estimates into more interpretable ones, the simulation resulted in a very complex scenario that was itself difficult to interpret.

This study has examined a particular approach to simulation, but other strategies may ultimately prove to be more effective at increasing the interpretability of estimates made from incomplete data sources. On the other hand, the results highlight some inherent limitations that are likely to apply to various secondary data sources. For example, the weeks depressed in past year variable may be inaccurate because of faulty recall. As a result, the lack of statistical significance of some potential predictors of this outcome may be partially due to a dilution of their effects through non-differential misclassification bias. A bias of this sort may have contributed to the need for an extremely complex equation (see Table 5) describing determinants of recurrence because variation in the frequency of consecutive episodes ended up being attributed in the simulation to recurrence as opposed to persistence. Another limitation the NPHS is that a variable that is correlated with episode duration, severity, could not be included in the study because a severity measure was not incorporated in the survey. In addition, the model itself includes certain assumptions that could affect its outcome. One of these is the random assignment of an onset date for new episodes. This assumption may not be true. For example, there may be a seasonal variation in depressive episode incidence.

The analysis highlights an important aspect of the longitudinal epidemiology of MD: the declining recovery rate as a function of time. According to DSM-IV TR [12] an untreated episode of MD "typically lasts four months or longer." The pattern of recovery, however, is such that most episodes are expected to be more brief than this whereas a smaller proportion are likely to be much longer - this distinction highlights the value of developing algorithms for predicting the course of MD and highlights the likely importance of symptom duration in addition to other predictive variables in anticipating the course of major depressive episodes. In other words, whereas the analysis indicates that older people tend to have longer episodes, a young person with an episode that has already lasted several months is likely to have a more negative prognosis than an older person with an episode that started more recently.

If the NPHS had fully recorded the duration of episodes, it would have been possible to model the pattern of recovery using conventional methods. Also, if the NPHS had directly measured the probability of recurrence among those respondents recovering between cycles, conventional approaches could have been used to model recurrence probabilities without the need for simulation. Such detailed longitudinal data are not available in most countries whereas less complete data often are available. As the epidemiology of MDE is further clarified, simulation may offer opportunities to improve the interpretability of readily available but methodologically limited data sources. However, an effective approach for accomplishing this goal has yet to be identified.

## List of Abbreviations

CIDI-SFMD: Composite International Diagnostic Interview Short Form for Major Depression; MDE: Major Depressive Episode; NPHS: National Population Health Survey.

## Competing interests

SP has received speaking fees from Lundbeck and consulting fees from Servier Canada. SP has also received consulting fees and has served on a Data Safety Monitoring Board for Cipher Pharmaceuticals, and has received a research grant from Servier Canada.

## Authors' contributions

SP is the sole author of the paper and was responsible for all aspect of the design and conduct of the study.

## Supplementary Material

Additional file 1 **Predicted episode durations**. Click here for file
