# Supplementary material for: Problems encountered with the use of simulation in an attempt to enhance interpretation of a secondary data source in epidemiologic mental health research
Source: BMC Res Notes. 2010 Aug 26;3:231. doi: 10.1186/1756-0500-3-231 (PMC2941757; doi:10.1186/1756-0500-3-231)

Supplemental File 1:

Predicted Episode Durations

Table of Contents:

Figure 1. Age 12-18, Pain, Smoking and Childhood Stressors 4
Figure 2. Age 12-18, Pain, Smoking and No Childhood Stressors 5
Figure 3. Age 12-18, Pain, No Smoking and Childhood Stressors 6
Figure 4. Age 12-18, Pain, No Smoking and No Childhood Stressors 7
Figure 5. Age 12-18, No Pain, Smoking and Childhood Stressors 8
Figure 6. Age 12-18, No Pain, Smoking and No Childhood Stressors 9
Figure 7. Age 12-18, No Pain, No Smoking and Childhood Stressors 10
Figure 8. Age 12-18, No Pain, No Smoking and No Childhood Stressors............11
Figure 9. Age 19-25, Pain, Smoking and Childhood Stressors 12
Figure 10. Age 19-25, Pain, Smoking and No Childhood Stressors.....................13
Figure 11. Age 19-25, Pain, No Smoking and Childhood Stressors.....................14
Figure 12. Age 19-25, Pain, No Smoking and No Childhood Stressors 15
Figure 13. Age 19-25, No Pain, Smoking and Childhood Stressors.....................16
Figure 14. Age 19-25, No Pain, Smoking and No Childhood Stressors 17
Figure 15. Age 19-25, No Pain, No Smoking and Childhood Stressors 18
Figure 16. Age 19-25, No Pain, No Smoking and No Childhood Stressors..........19
Figure 17. Age 26-45, Pain, Smoking and Childhood Stressors 20
Figure 18. Age 26-45, Pain, Smoking and No Childhood Stressors.....................21
Figure 19. Age 26-45, Pain, No Smoking and Childhood Stressors.....................22
Figure 20. Age 26-45, Pain, No Smoking and No Childhood Stressors 23
Figure 21. Age 26-45, No Pain, Smoking and Childhood Stressors.....................24
Figure 22. Age 26-45, No Pain, Smoking and No Childhood Stressors 25
Figure 23. Age 26-45, No Pain, No Smoking and Childhood Stressors 26
Figure 24. Age 26-45, No Pain, No Smoking and No Childhood Stressors..........27
Figure 25. Age 46-65, Pain, Smoking and Childhood Stressors 28

Figure 26. Age 46-65, Pain, Smoking and No Childhood Stressors....................29
Figure 27. Age 46-65, Pain, No Smoking and Childhood Stressors....................30
Figure 28. Age 46-65, Pain, No Smoking and No Childhood Stressors 31
Figure 29. Age 46-65, No Pain, Smoking and Childhood Stressors....................32
Figure 30. Age 46-65, No Pain, Smoking and No Childhood Stressors 33
Figure 31. Age 46-65, No Pain, No Smoking and Childhood Stressors 34
Figure 32. Age 46-65, No Pain, No Smoking and No Childhood Stressors..........35
Figure 33. Age 66 or more, Pain, Smoking and Childhood Stressors 36
Figure 34. Age 66 or more, Pain, Smoking and No Childhood Stressors 37
Figure 35. Age 66 or more, Pain, No Smoking and Childhood Stressors 38
Figure 36. Age 66 or more, Pain, No Smoking and No Childhood Stressors.......39
Figure 37. Age 66 or more, No Pain, Smoking and Childhood Stressors 40
Figure 38. Age 66 or more, No Pain, Smoking and No Childhood Stressors.......41
Figure 39. Age 66 or more, No Pain, No Smoking and Childhood Stressors.......42
Figure 40. Age 66 or more, No Pain, No Smoking and No Childhood Stressors .43

Figure 1. Age 12-18, Pain, Smoking and Childhood Stressors

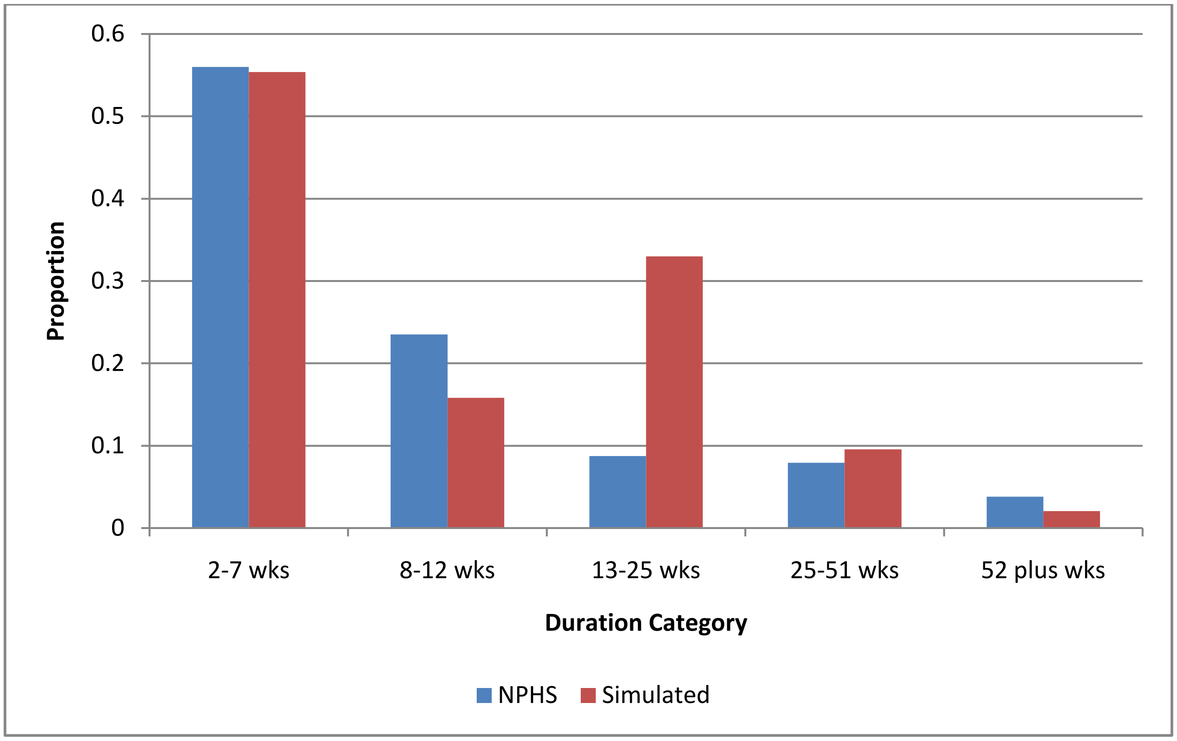

Figure 2. Age 12-18, Pain, Smoking and No Childhood Stressors

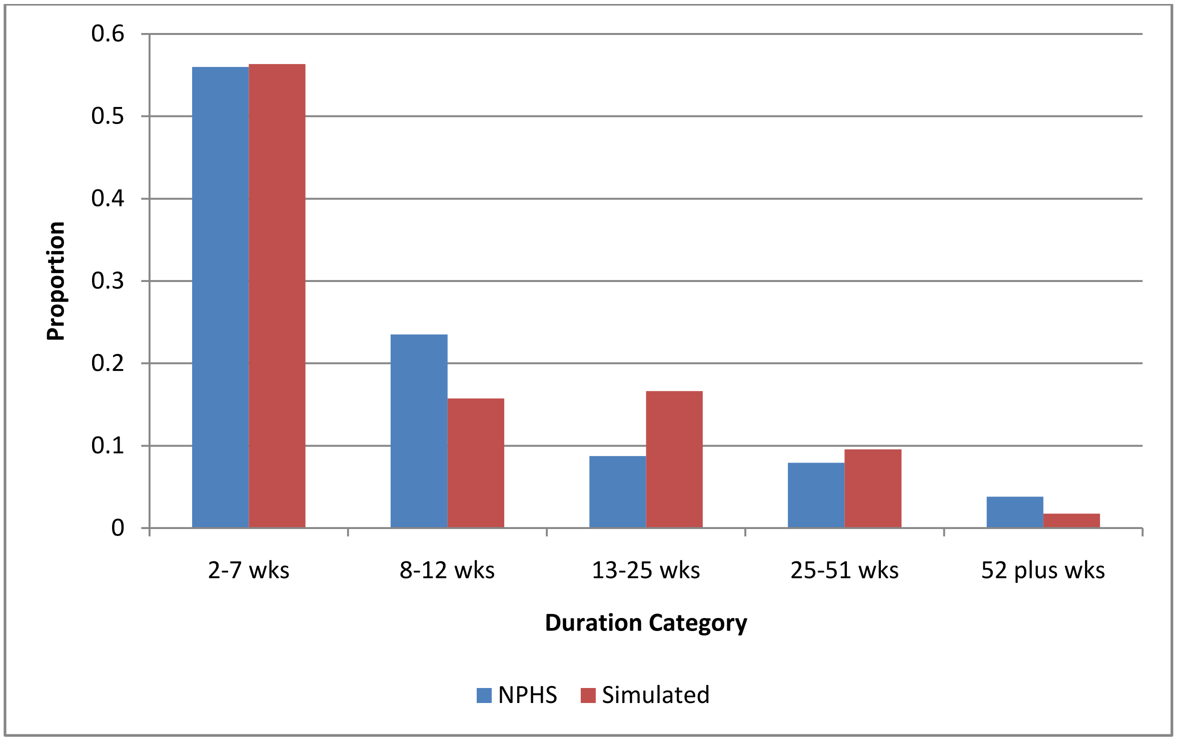

Figure 3. Age 12-18, Pain, No Smoking and Childhood Stressors

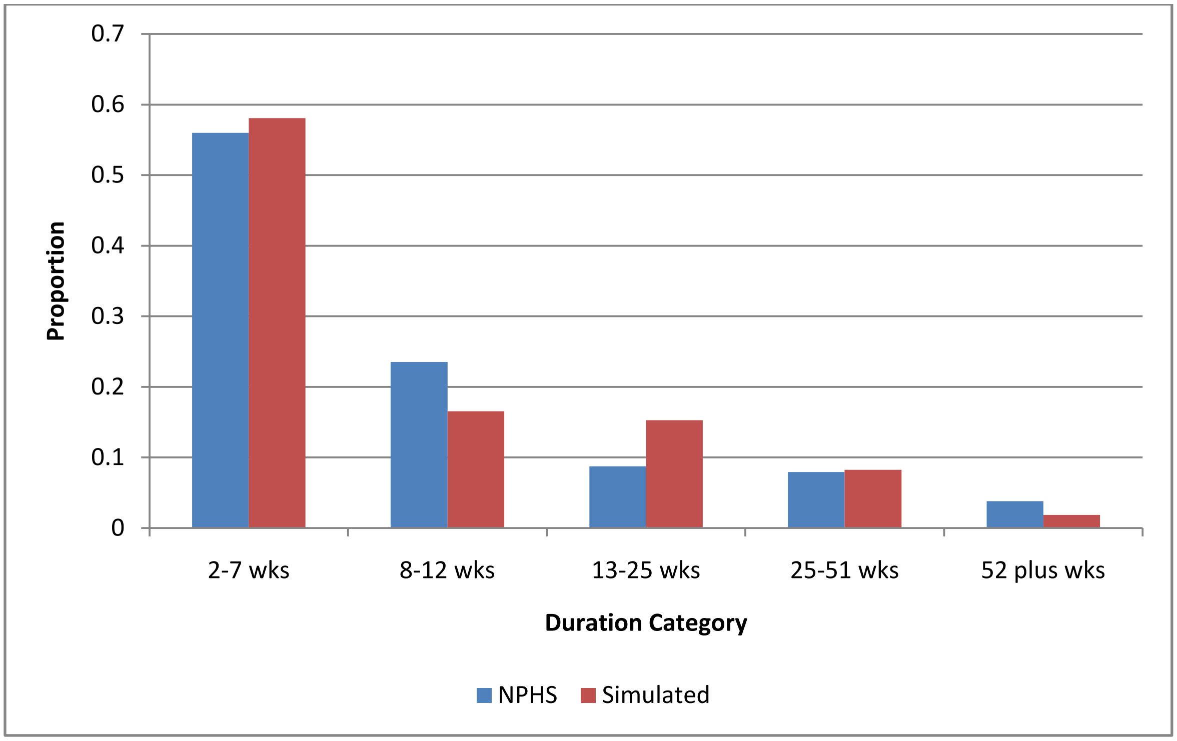

Figure 4. Age 12-18, Pain, No Smoking and No Childhood Stressors

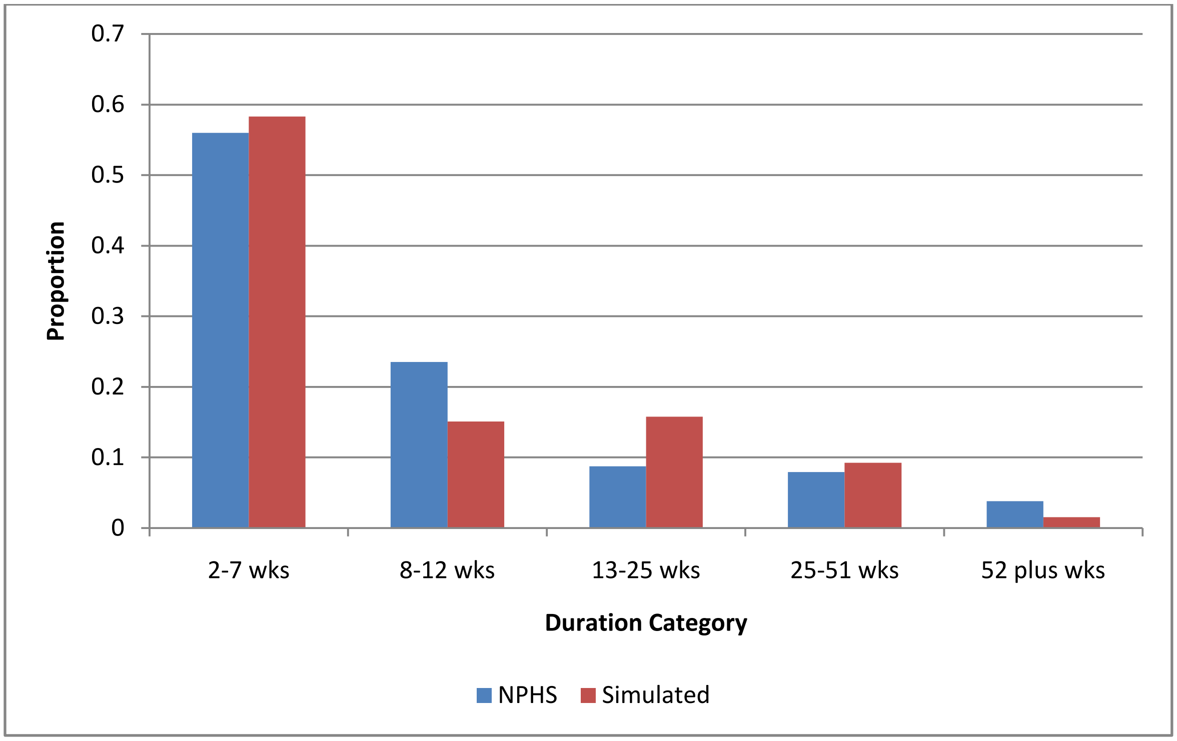

Figure 5. Age 12-18, No Pain, Smoking and Childhood Stressors

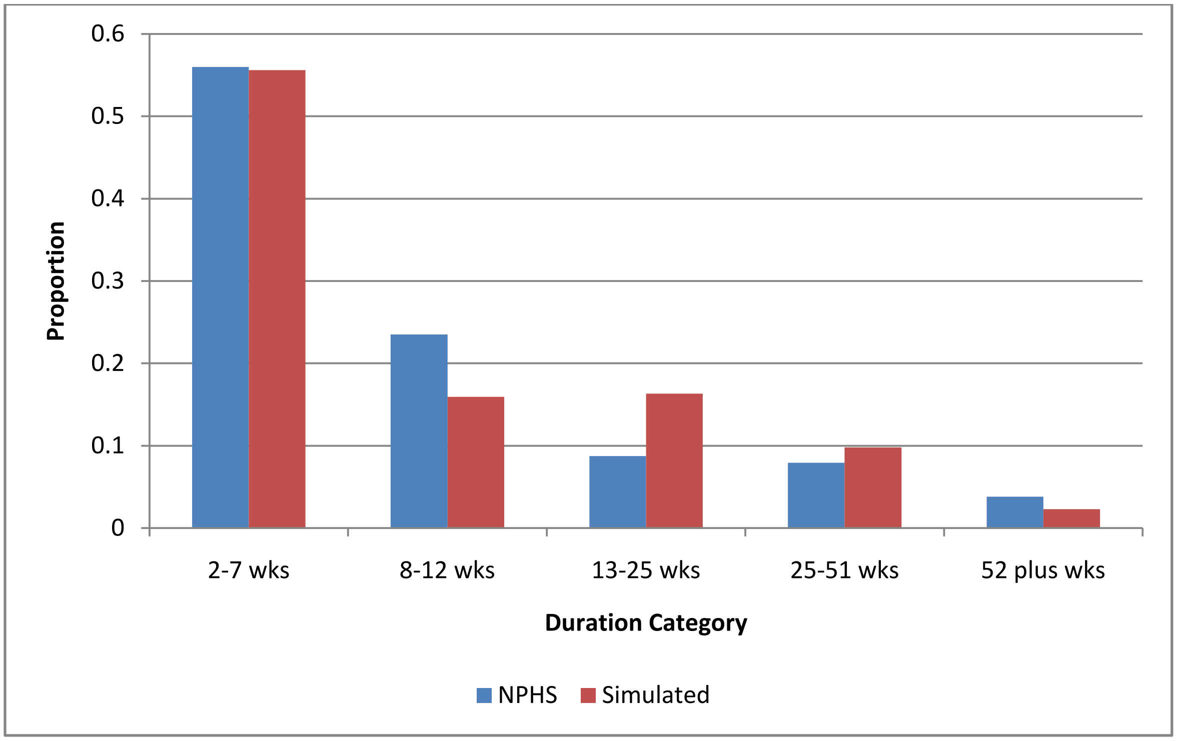

Figure 6. Age 12-18, No Pain, Smoking and No Childhood Stressors

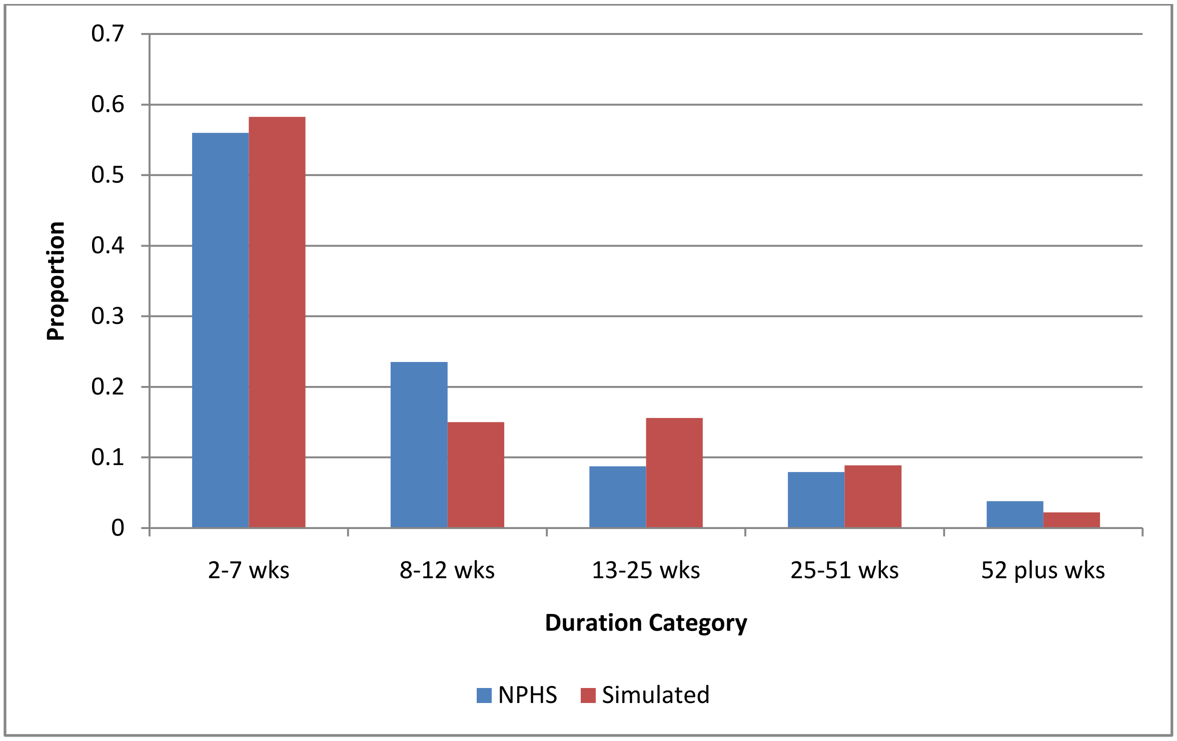

Figure 7. Age 12-18, No Pain, No Smoking and Childhood Stressors

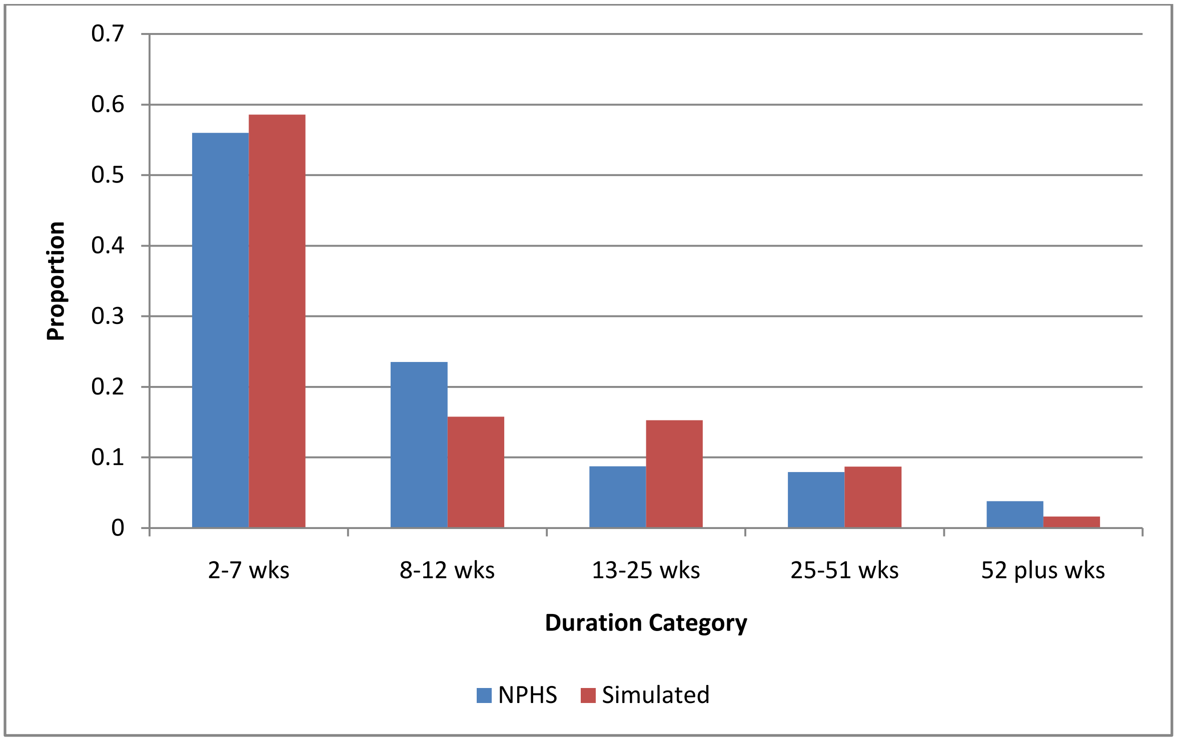

Figure 8. Age 12-18, No Pain, No Smoking and No Childhood Stressors

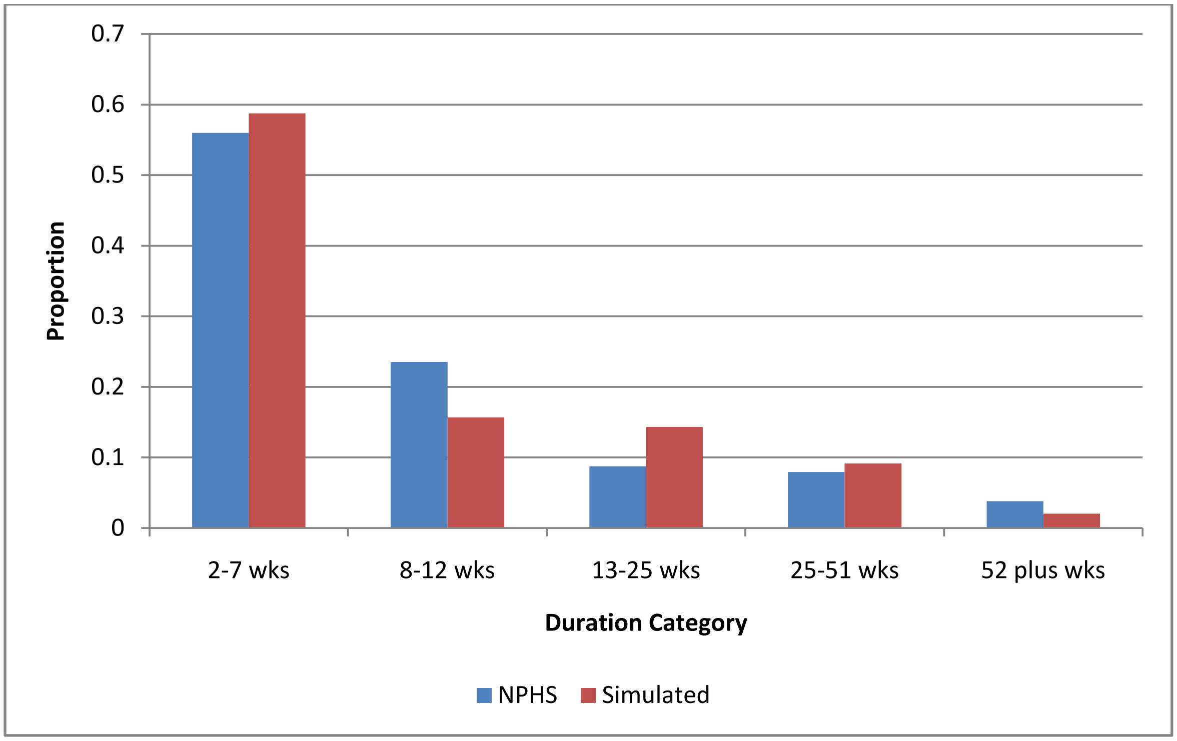

Figure 9. Age 19-25, Pain, Smoking and Childhood Stressors

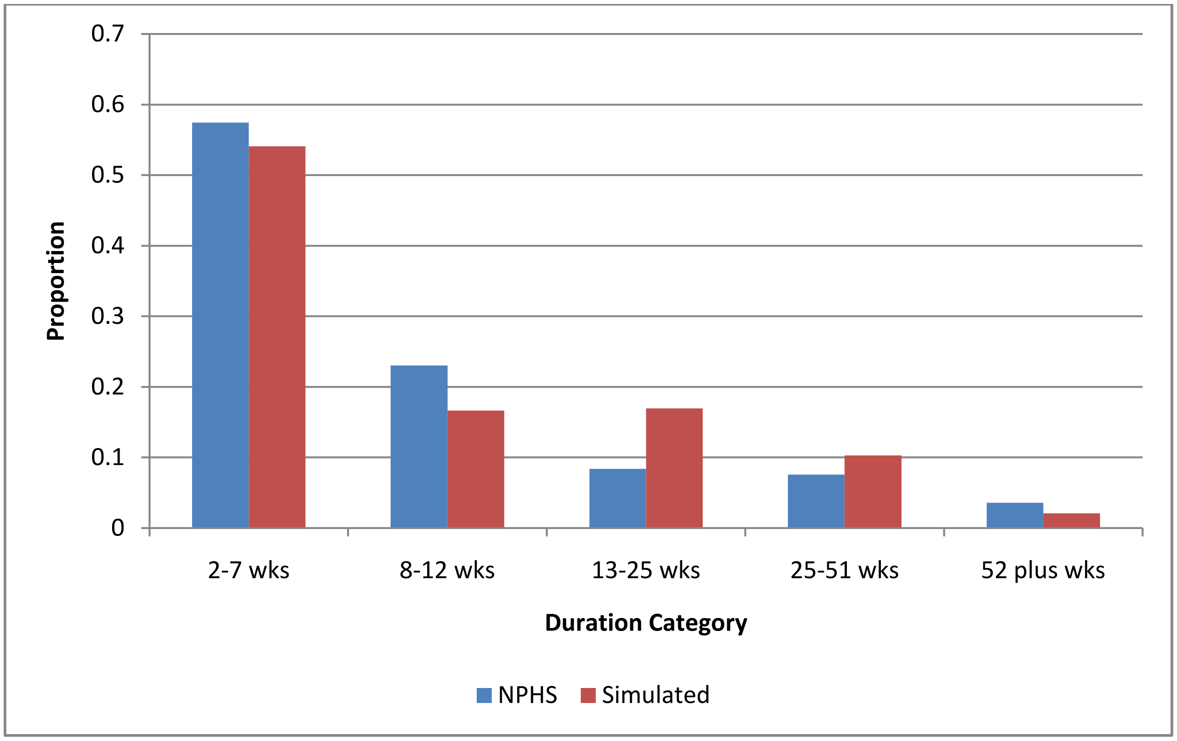

Figure 10. Age 19-25, Pain, Smoking and No Childhood Stressors

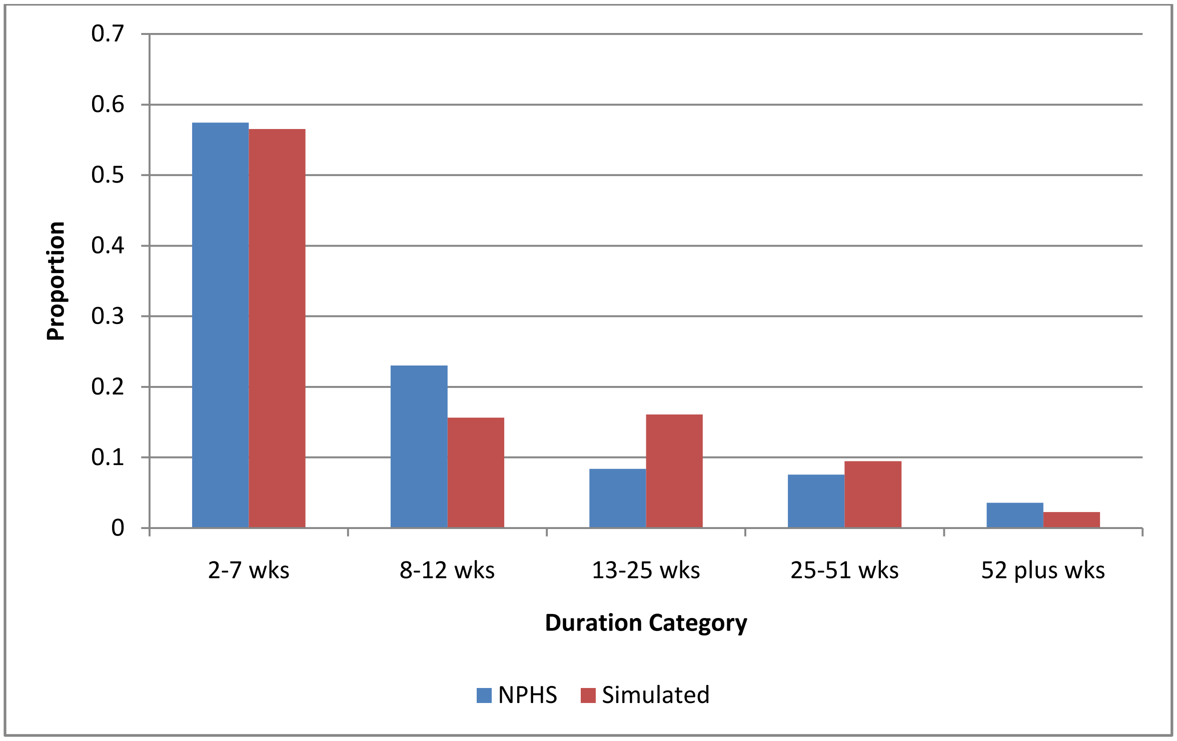

Figure 11. Age 19-25, Pain, No Smoking and Childhood Stressors

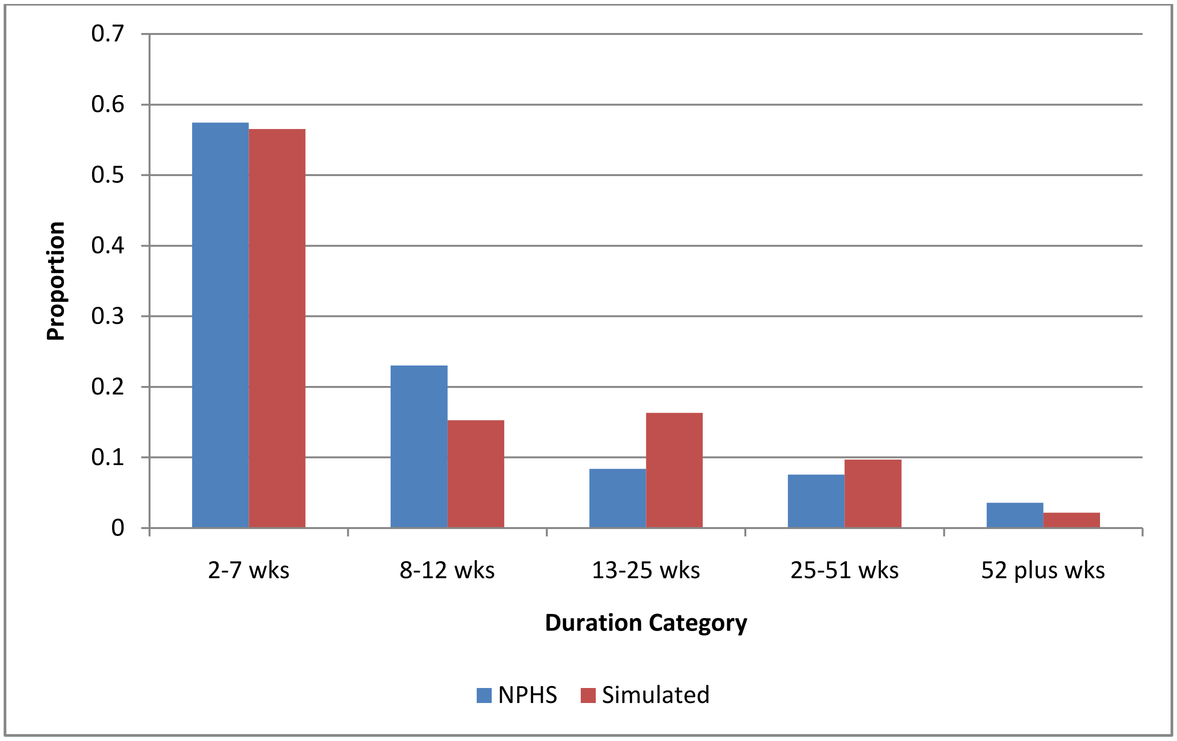

Figure 12. Age 19-25, Pain, No Smoking and No Childhood Stressors

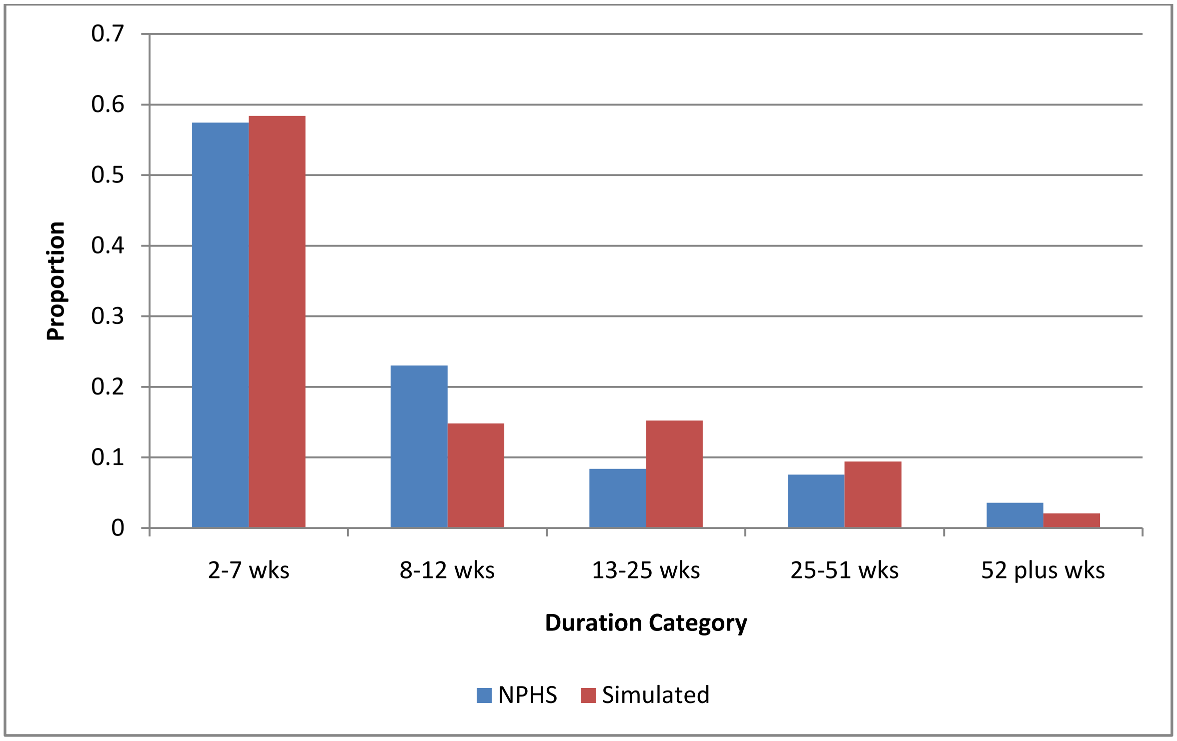

Figure 13. Age 19-25, No Pain, Smoking and Childhood Stressors

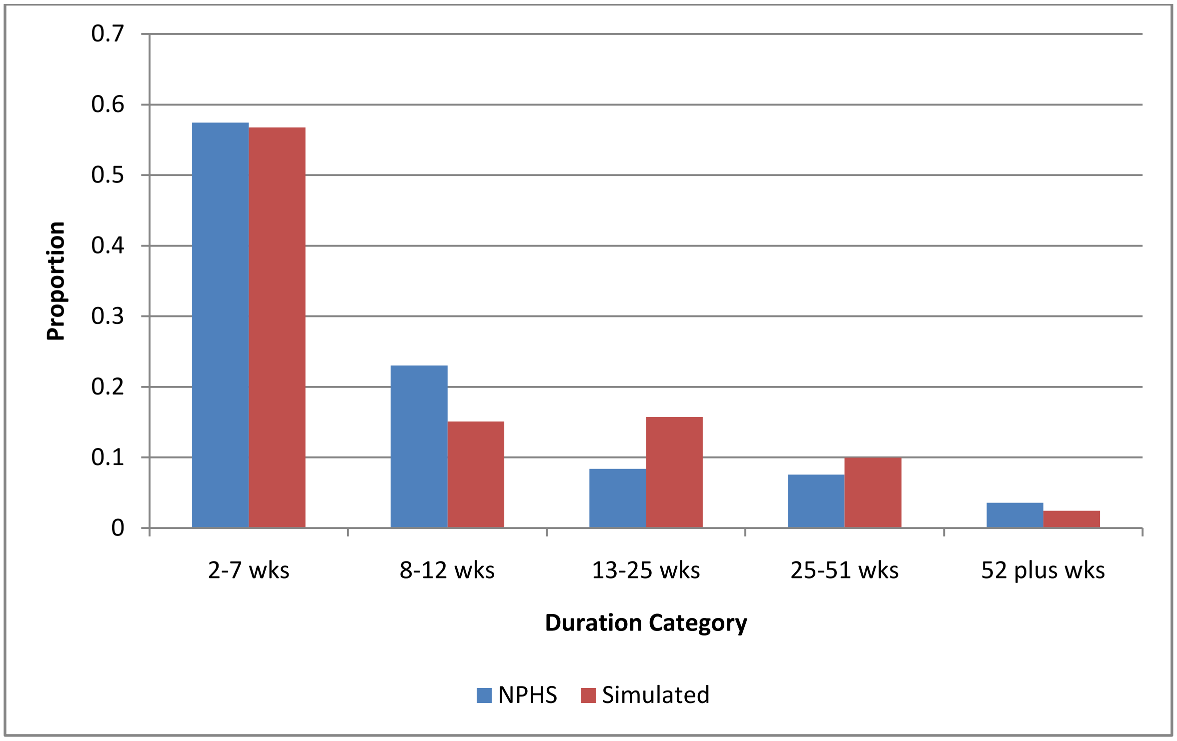

Figure 14. Age 19-25, No Pain, Smoking and No Childhood Stressors

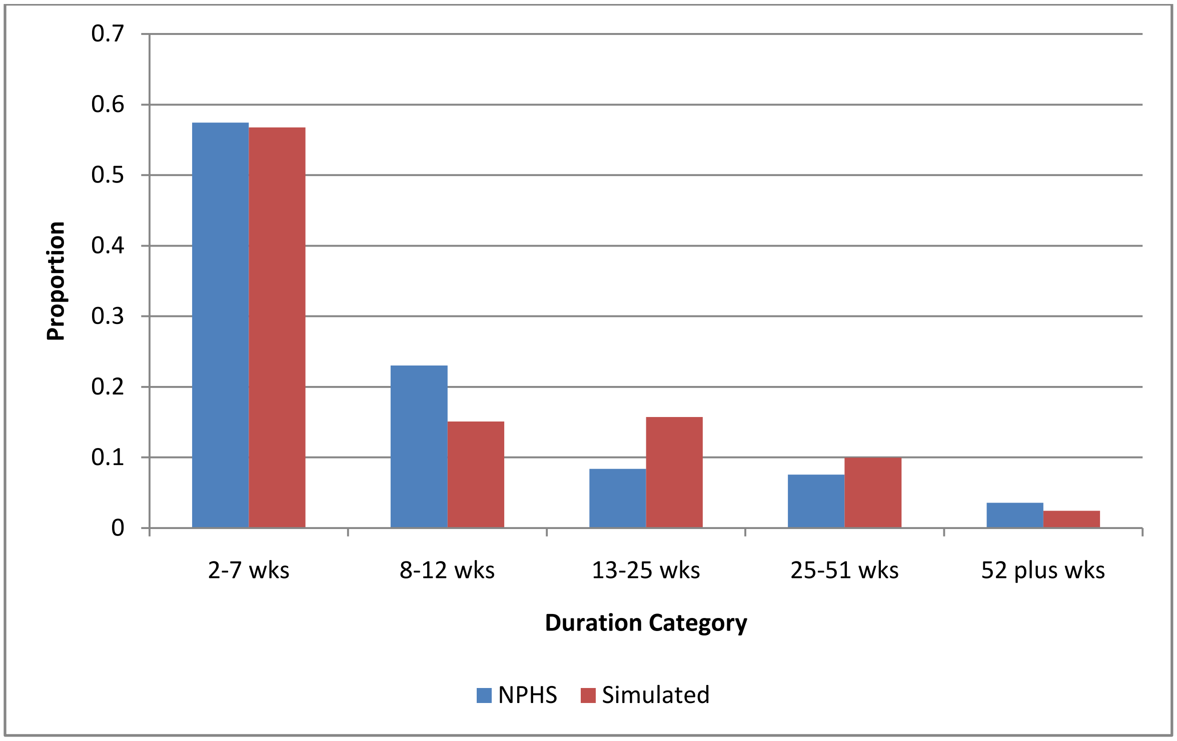

Figure 15. Age 19-25, No Pain, No Smoking and Childhood Stressors

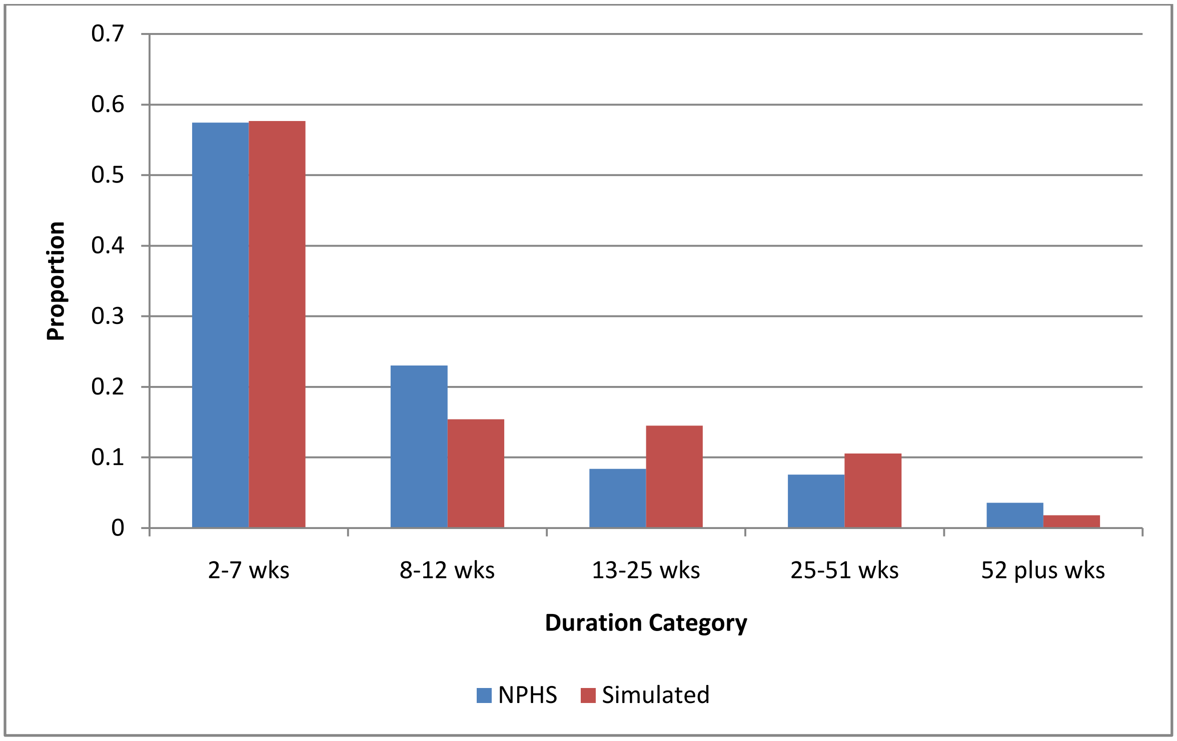

Figure 16. Age 19-25, No Pain, No Smoking and No Childhood Stressors

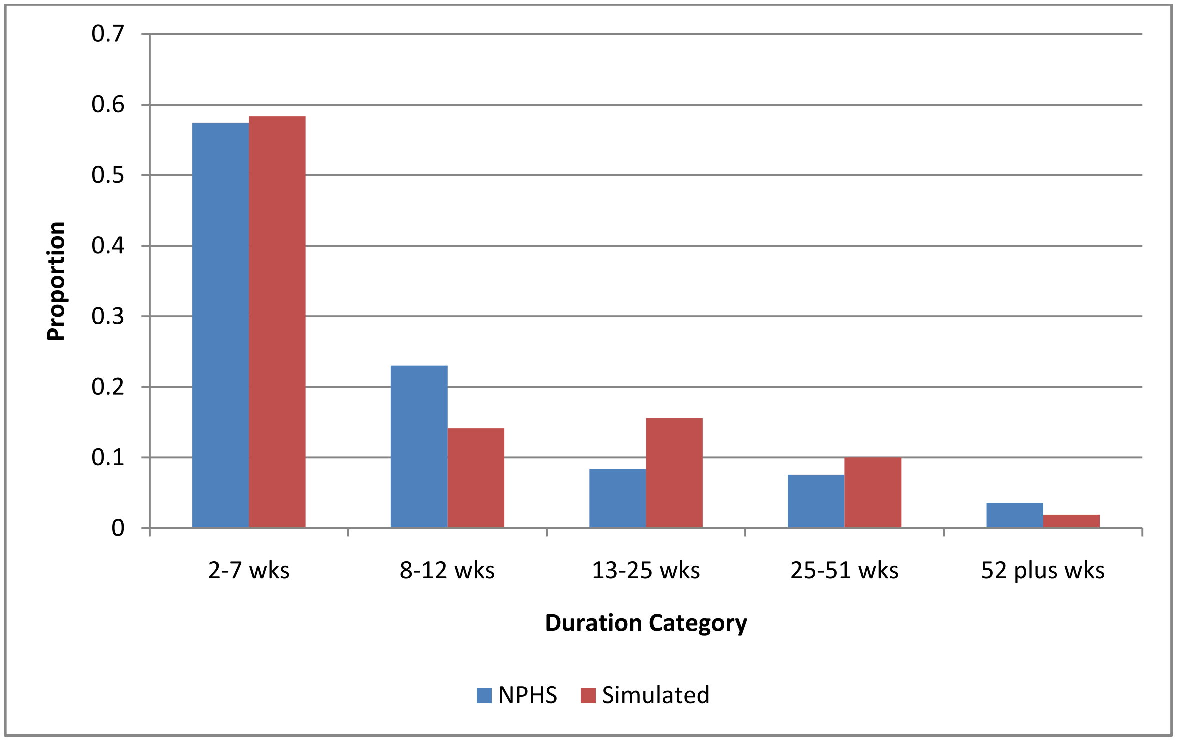

Figure 17. Age 26-45, Pain, Smoking and Childhood Stressors

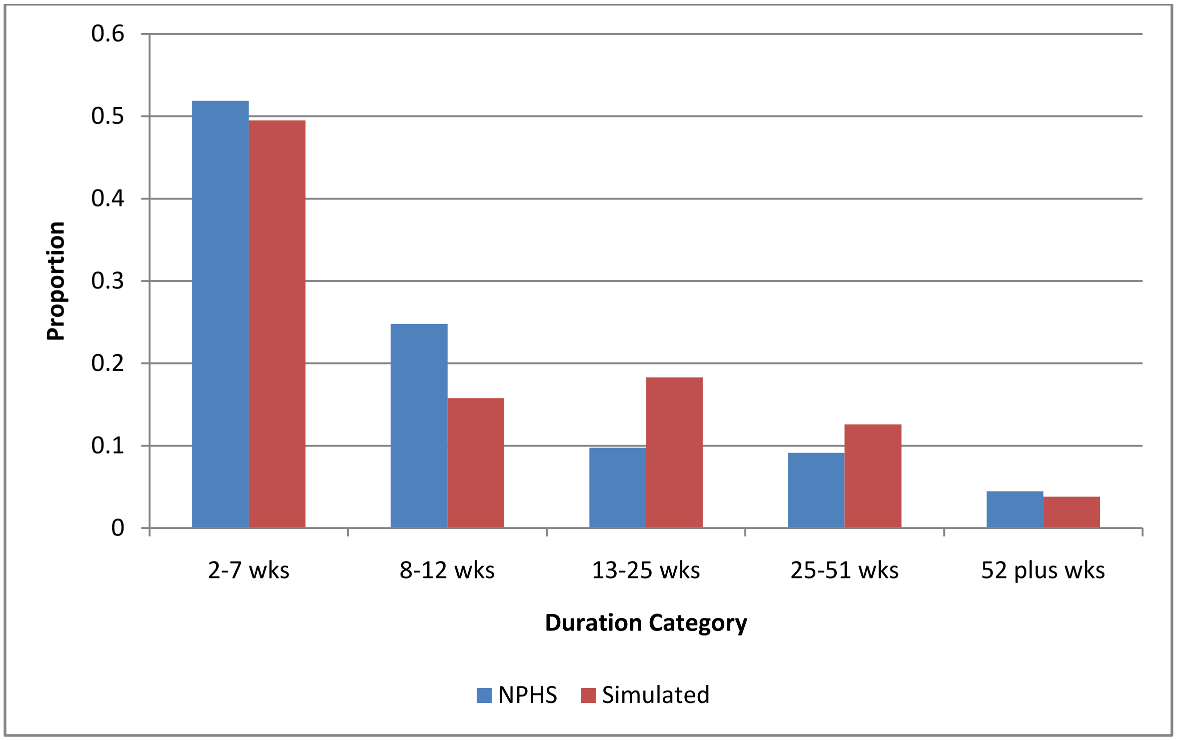

Figure 18. Age 26-45, Pain, Smoking and No Childhood Stressors

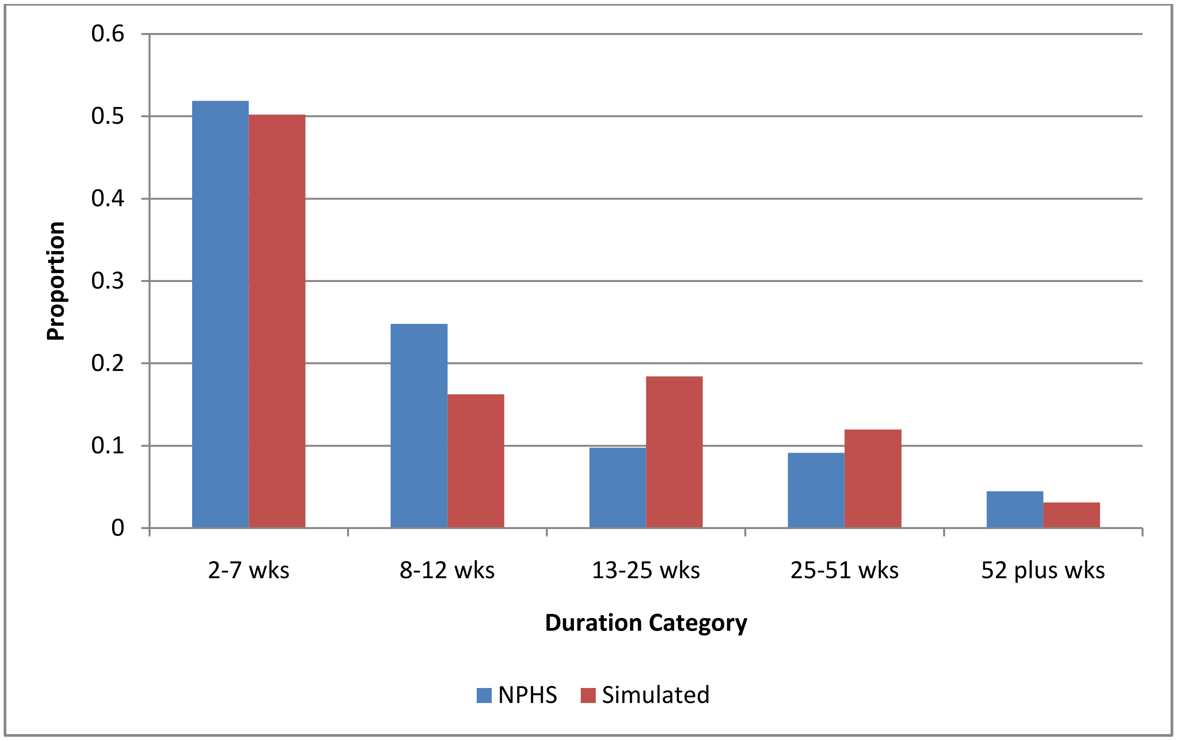

Figure 19. Age 26-45, Pain, No Smoking and Childhood Stressors

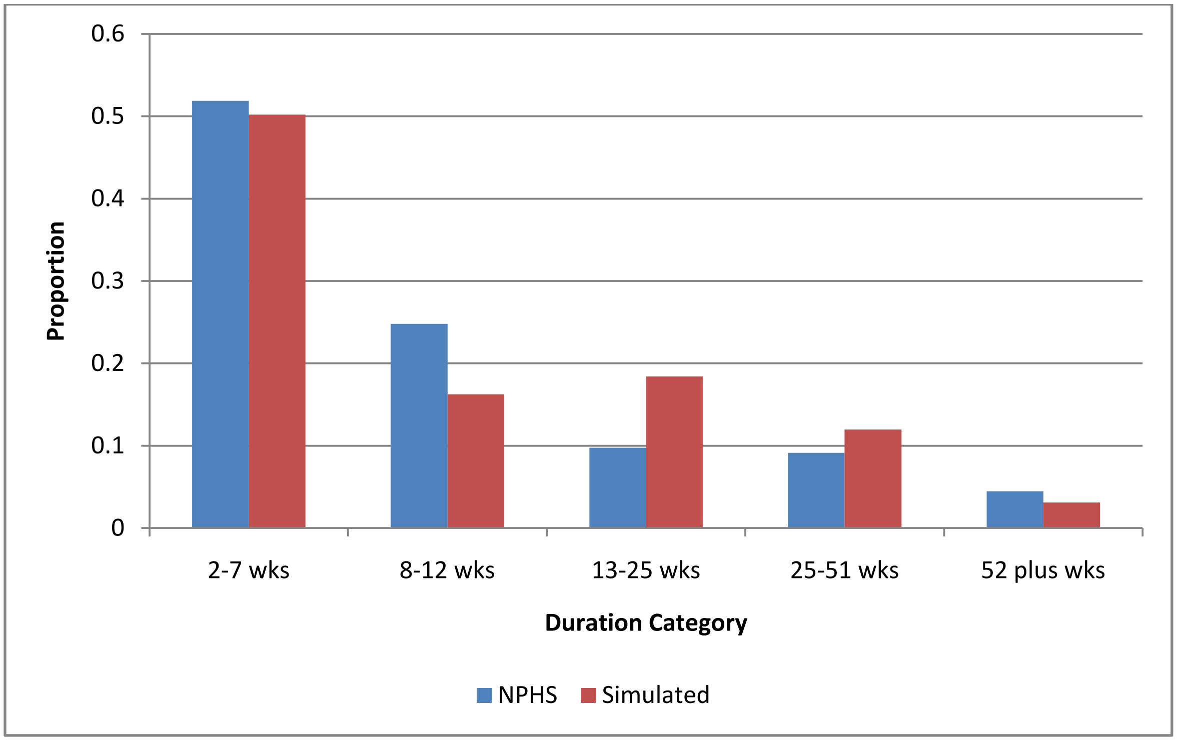

Figure 20. Age 26-45, Pain, No Smoking and No Childhood Stressors

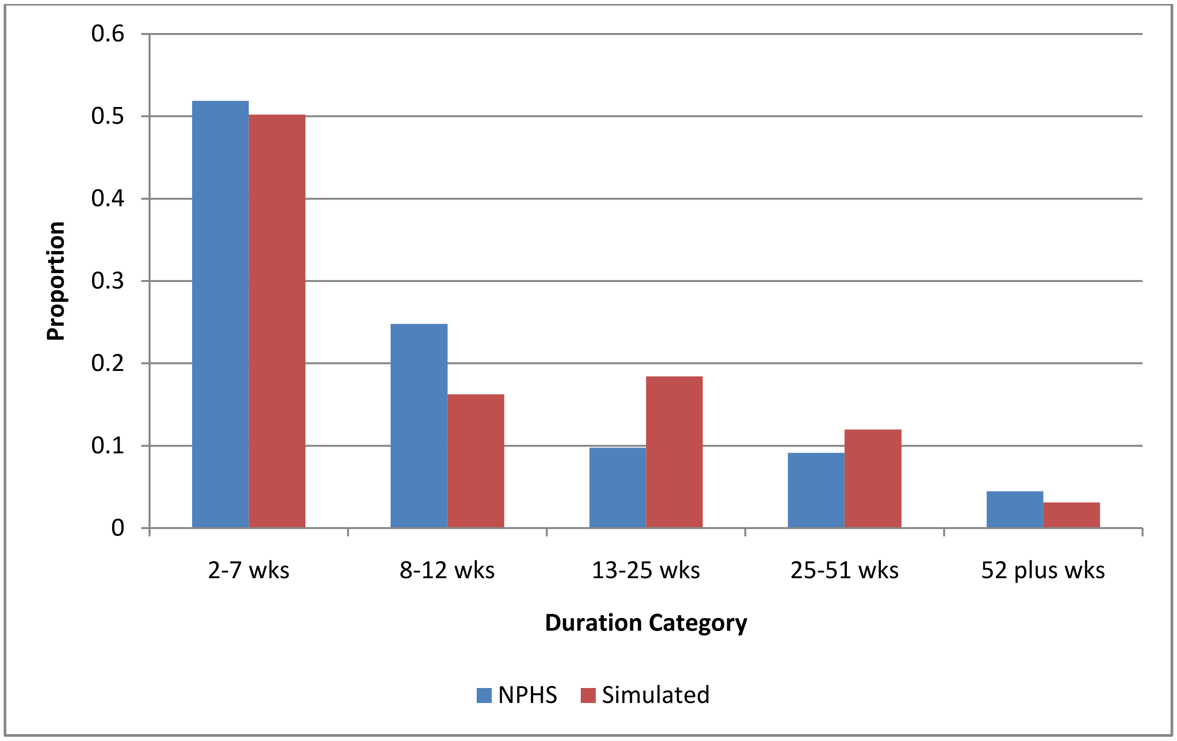

Figure 21. Age 26-45, No Pain, Smoking and Childhood Stressors

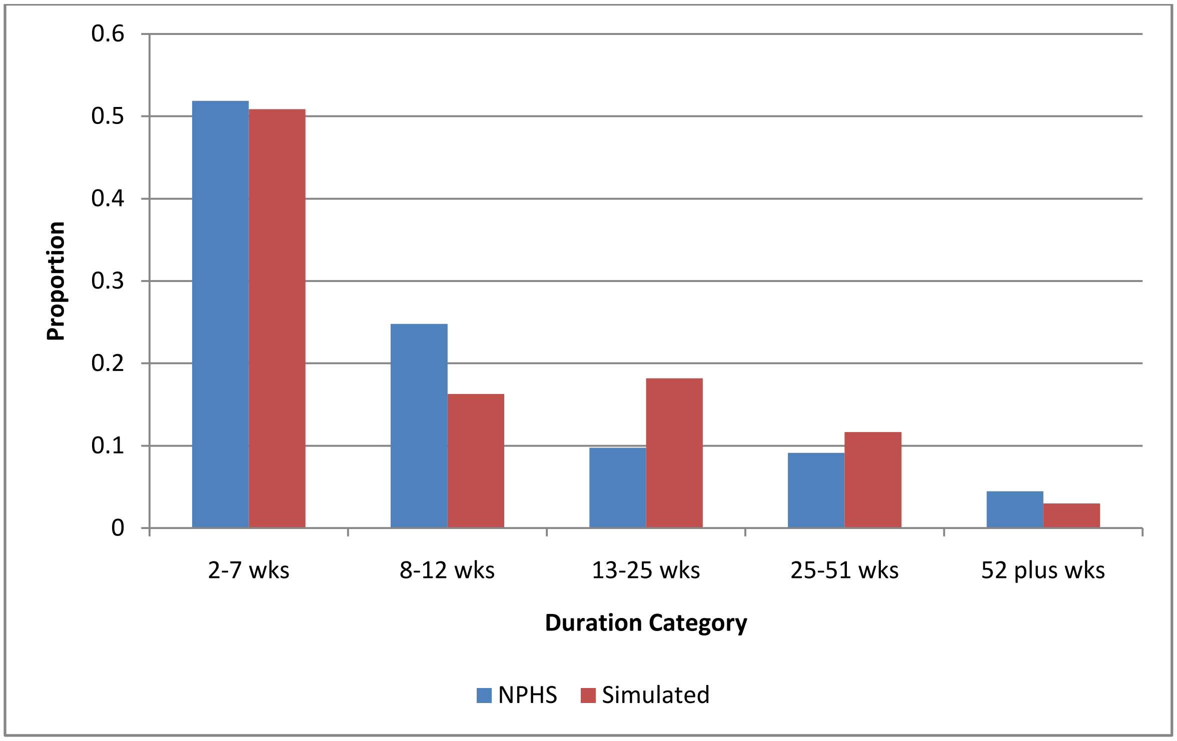

Figure 22. Age 26-45, No Pain, Smoking and No Childhood Stressors

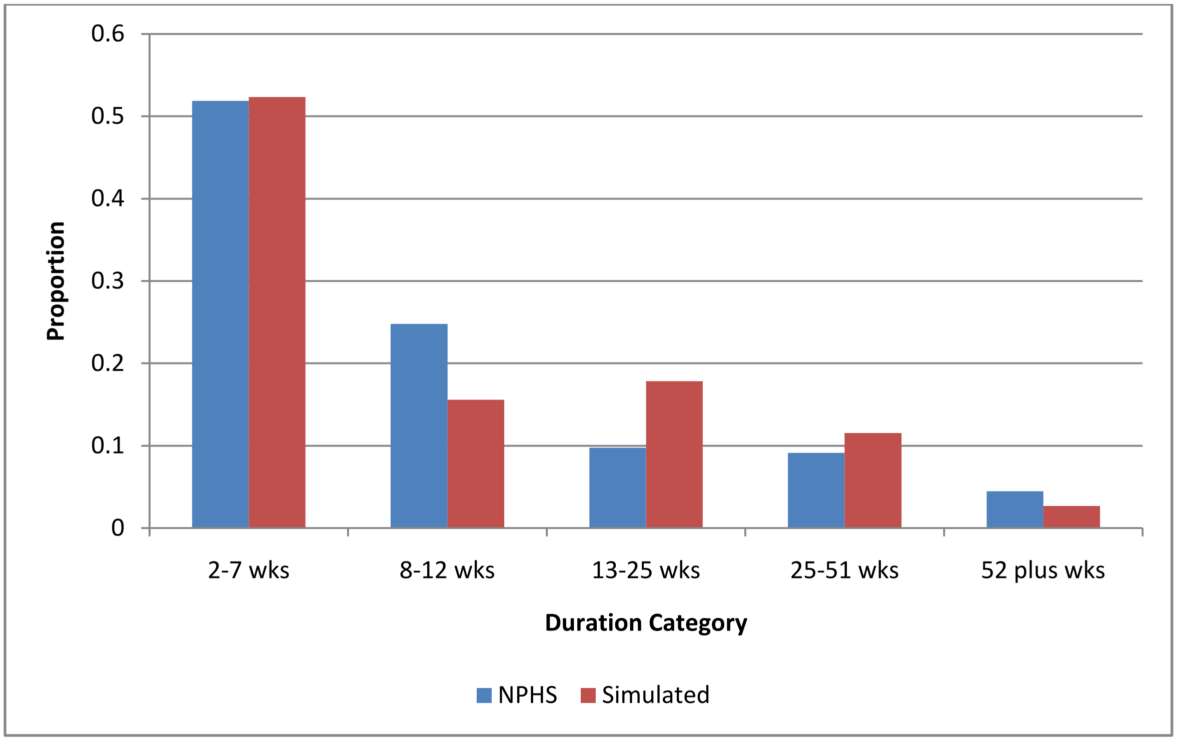

Figure 23. Age 26-45, No Pain, No Smoking and Childhood Stressors

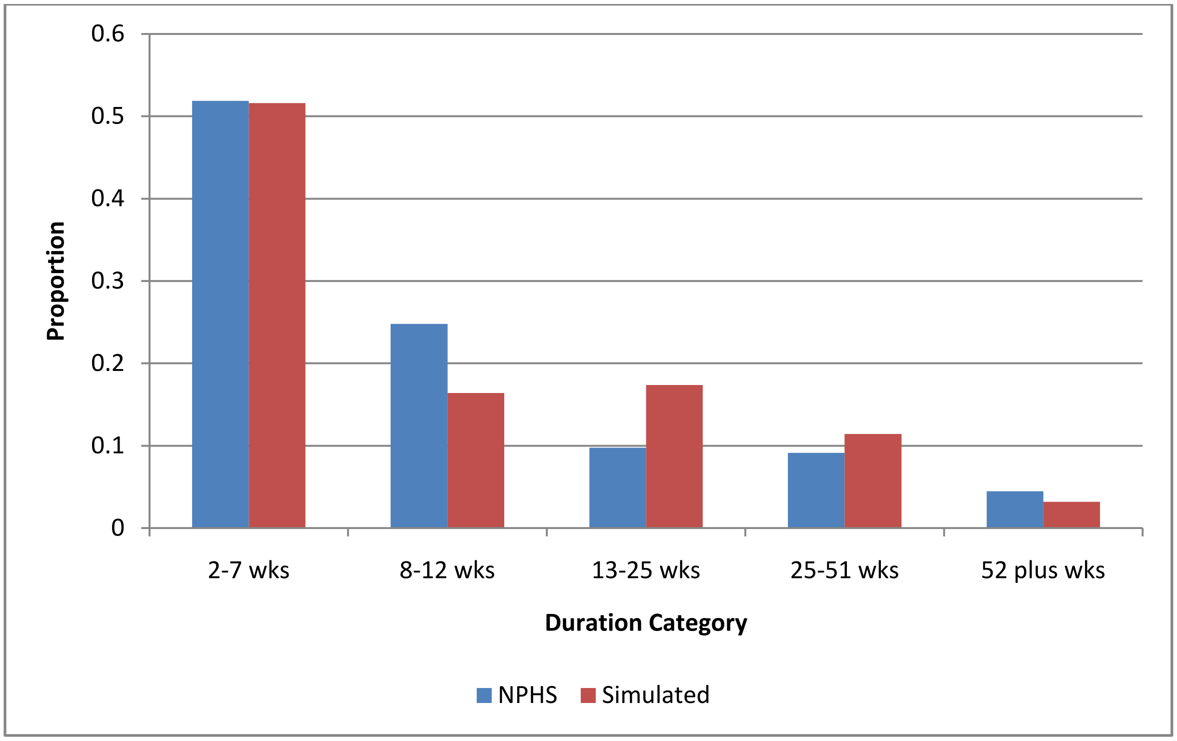

Figure 24. Age 26-45, No Pain, No Smoking and No Childhood Stressors

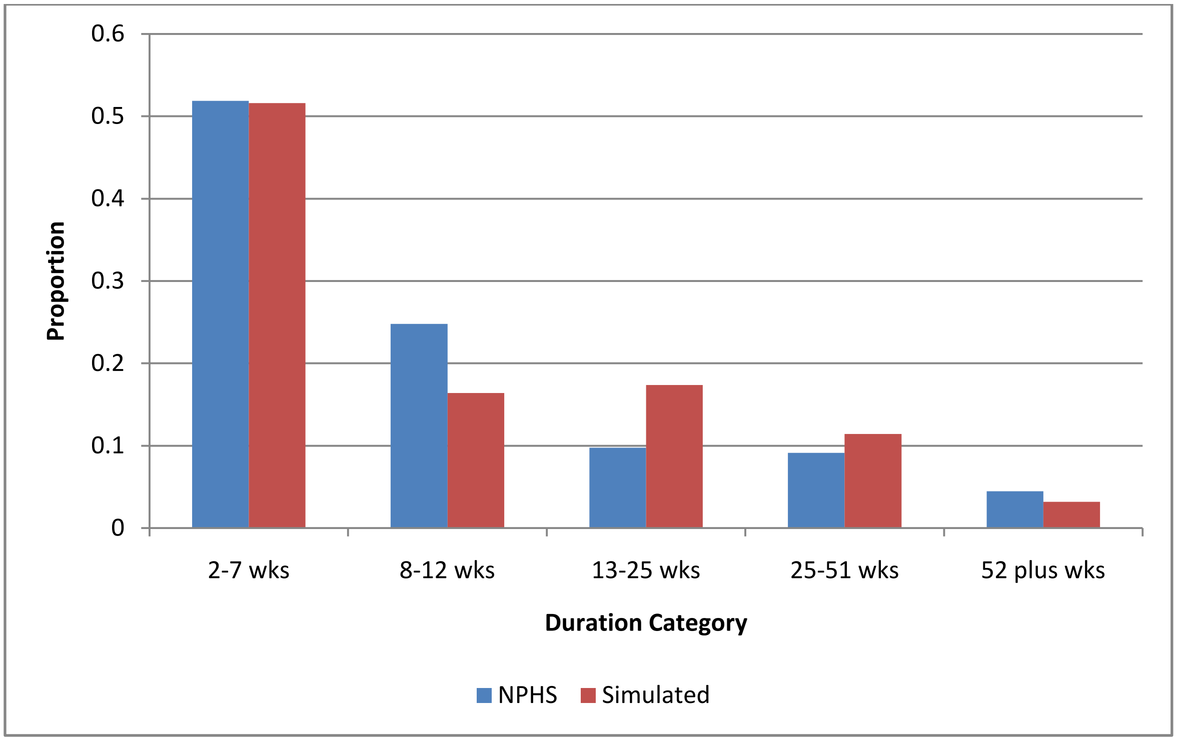

Figure 25. Age 46-65, Pain, Smoking and Childhood Stressors

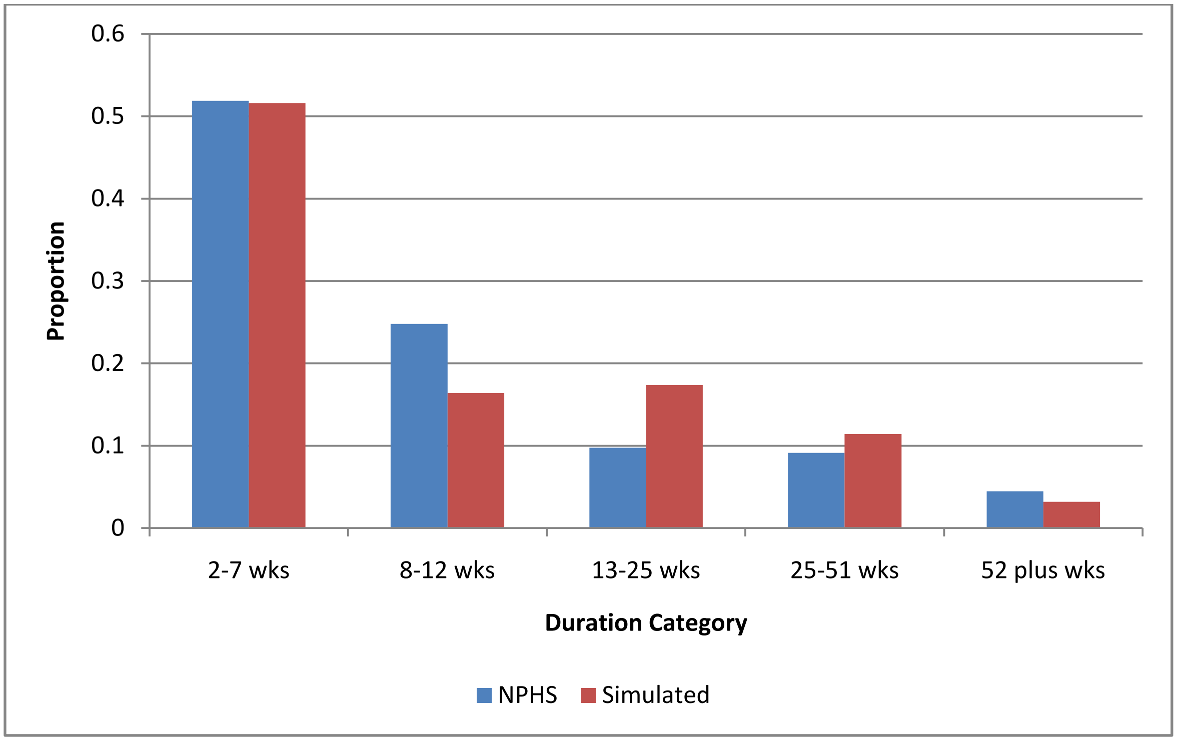

Figure 26. Age 46-65, Pain, Smoking and No Childhood Stressors

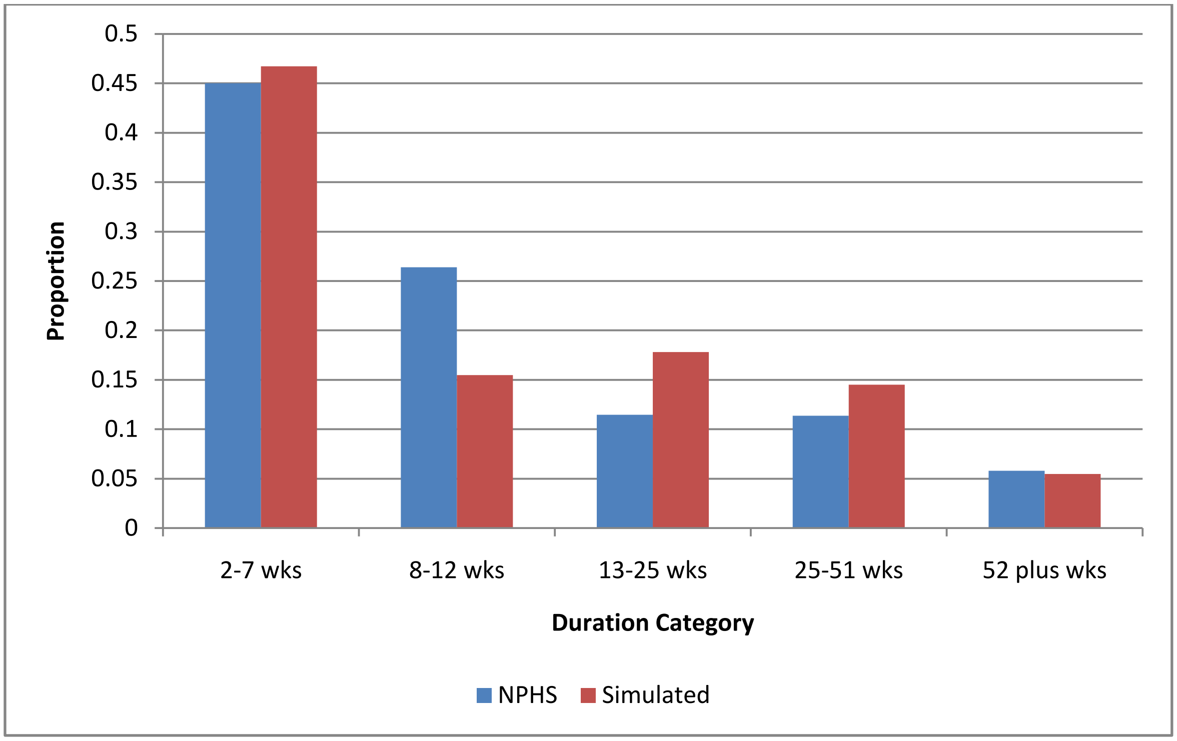

Figure 27. Age 46-65, Pain, No Smoking and Childhood Stressors

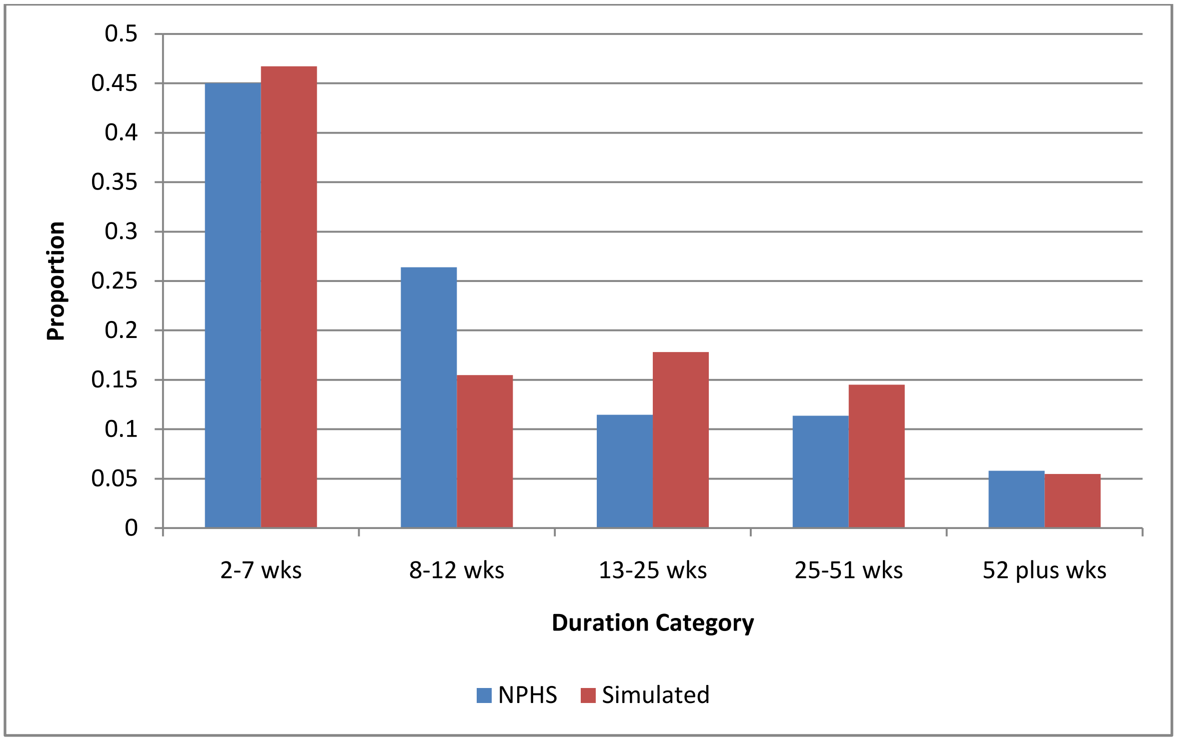

Figure 28. Age 46-65, Pain, No Smoking and No Childhood Stressors

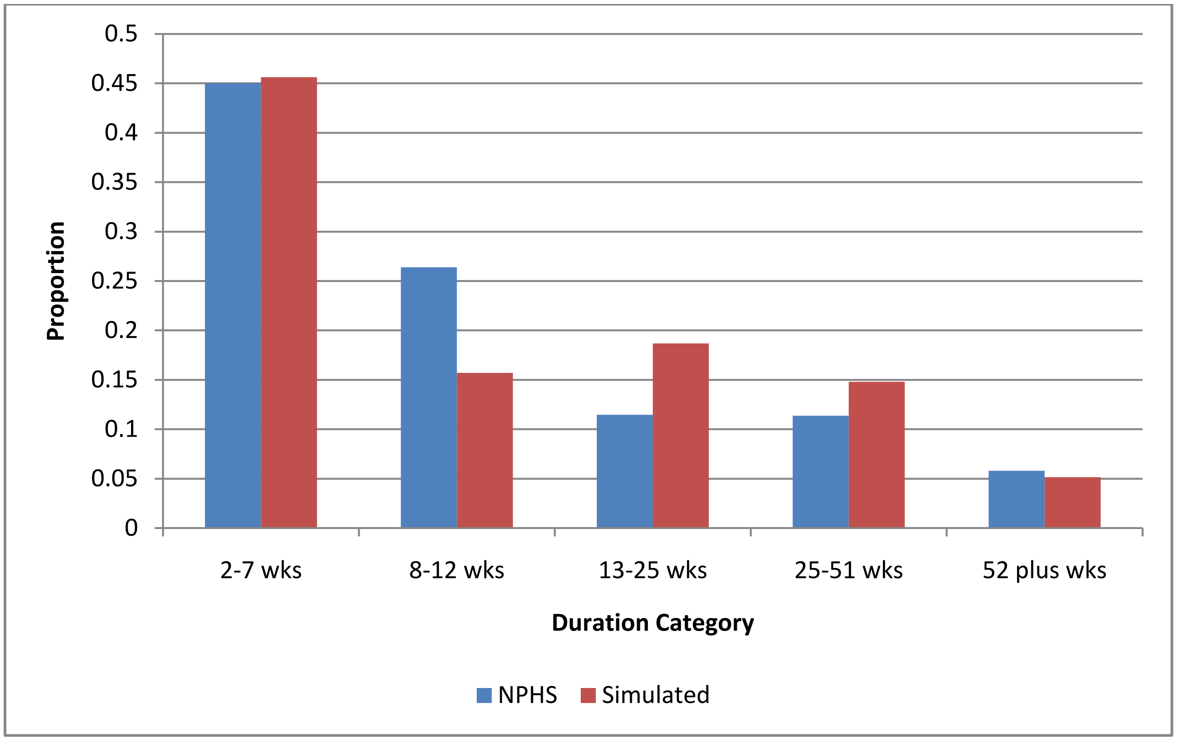

Figure 29. Age 46-65, No Pain, Smoking and Childhood Stressors

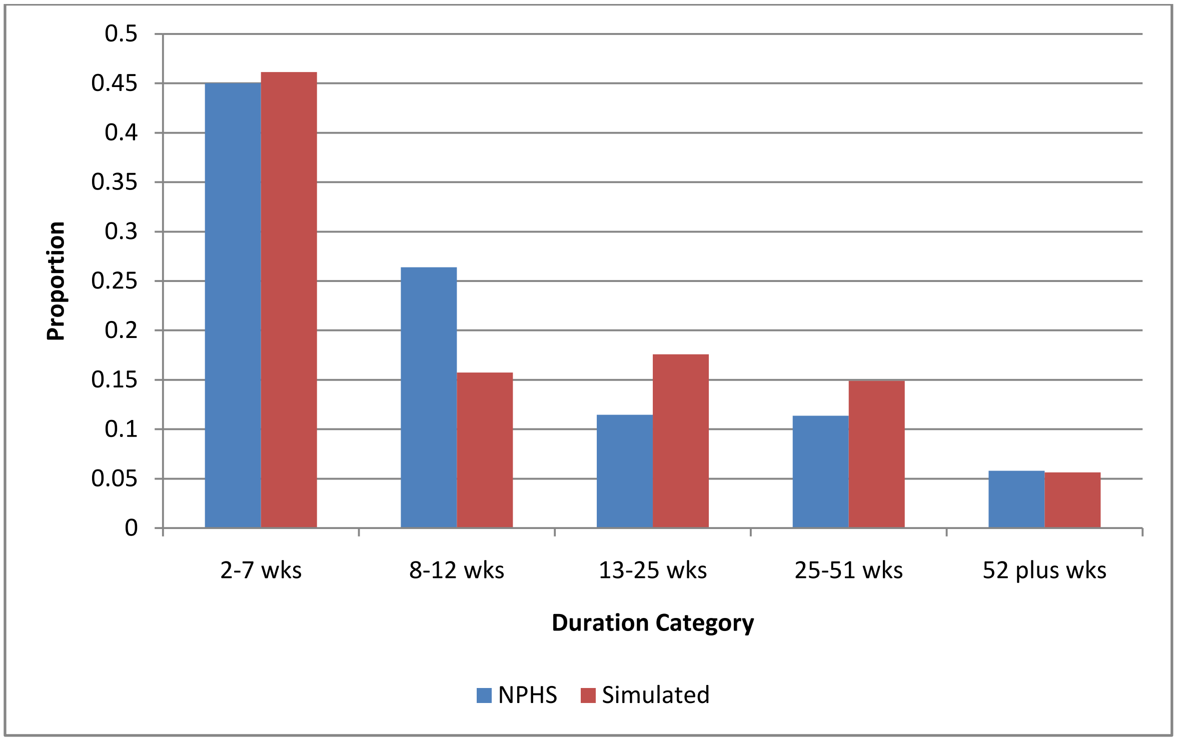

Figure 30. Age 46-65, No Pain, Smoking and No Childhood Stressors

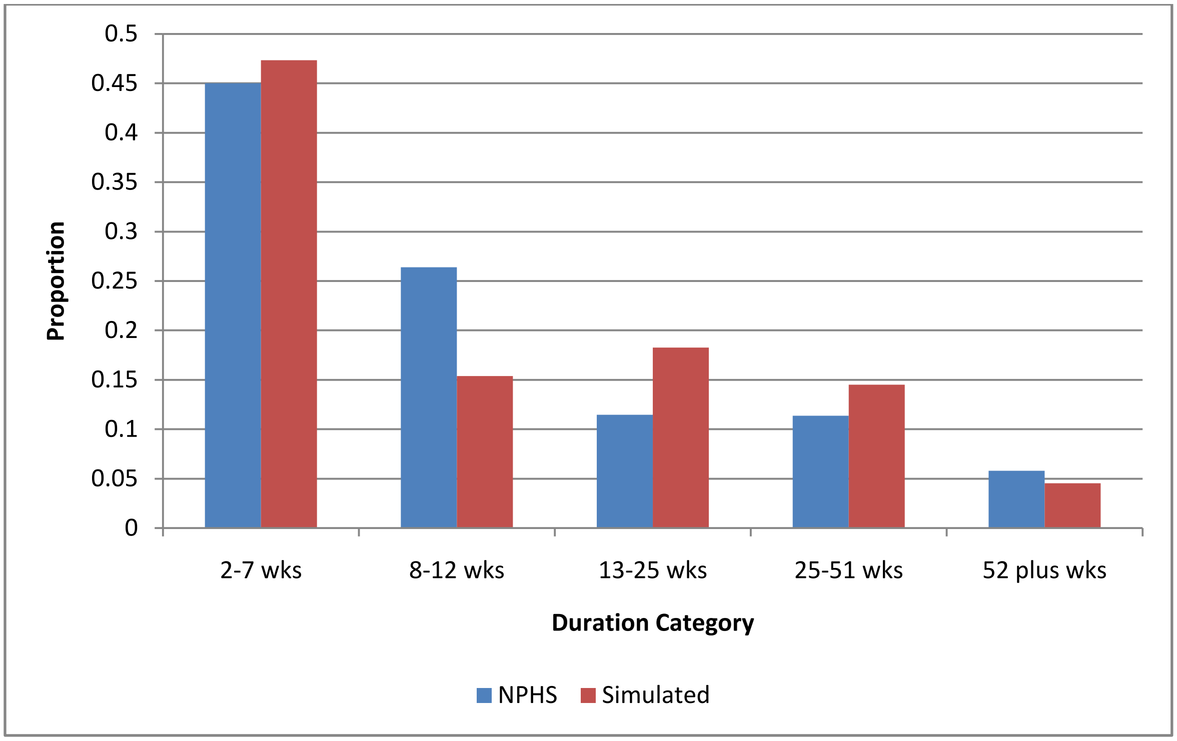

Figure 31. Age 46-65, No Pain, No Smoking and Childhood Stressors

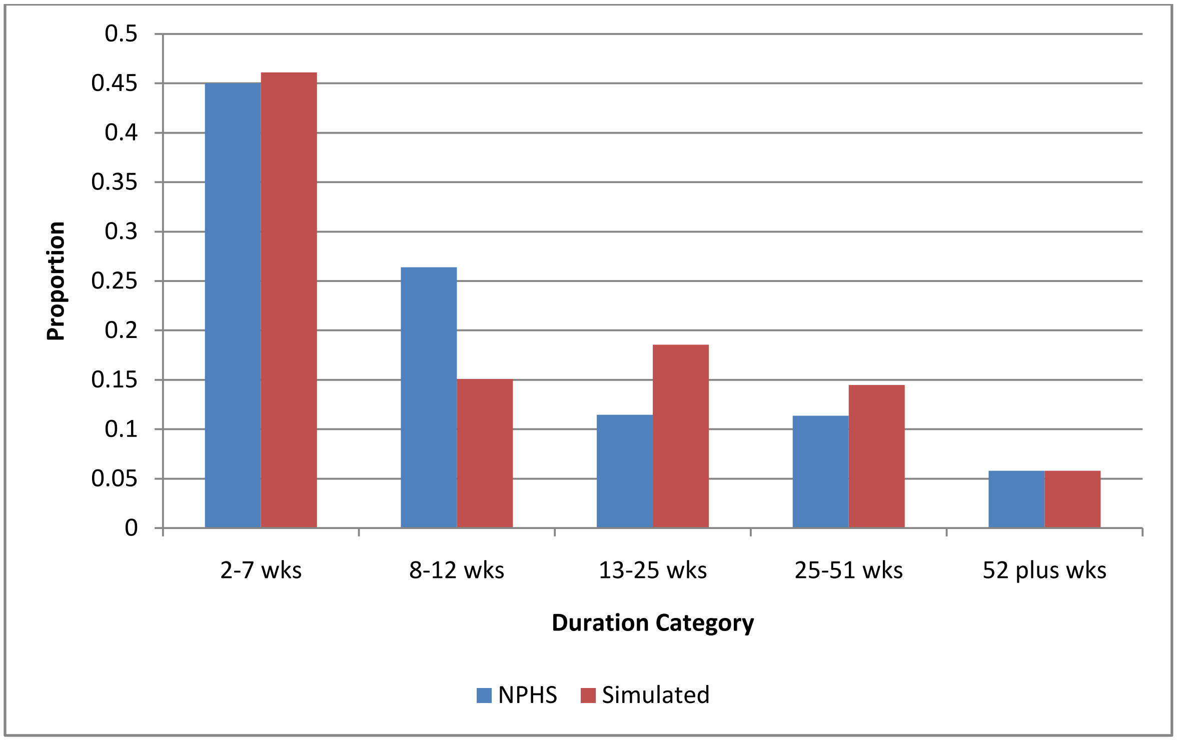

Figure 32. Age 46-65, No Pain, No Smoking and No Childhood Stressors

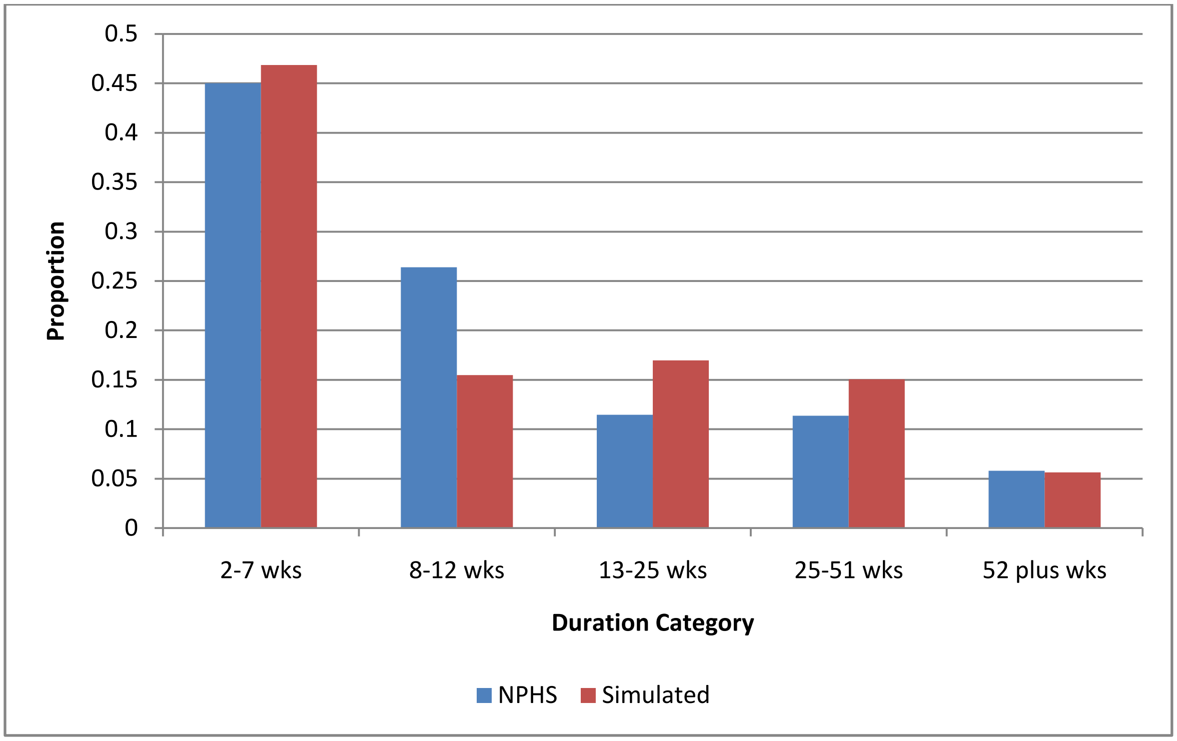

Figure 33. Age 66 or more, Pain, Smoking and Childhood Stressors

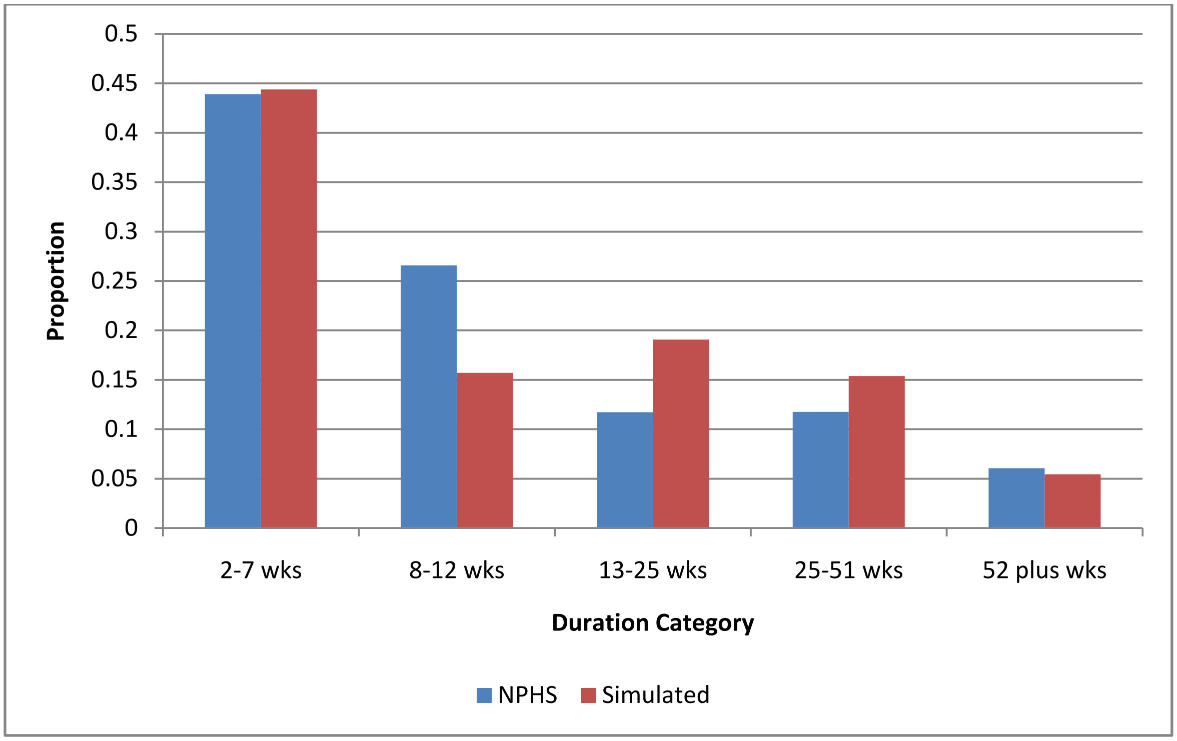

Figure 34. Age 66 or more, Pain, Smoking and No Childhood Stressors

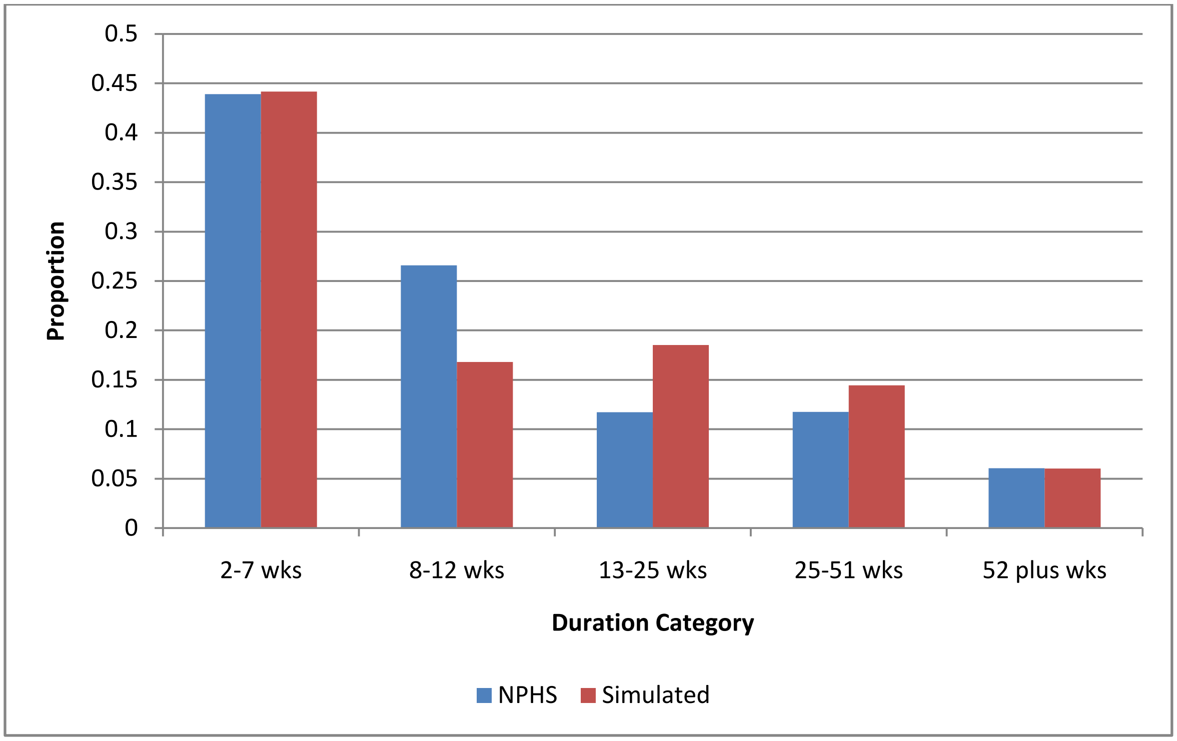

Figure 35. Age 66 or more, Pain, No Smoking and Childhood Stressors

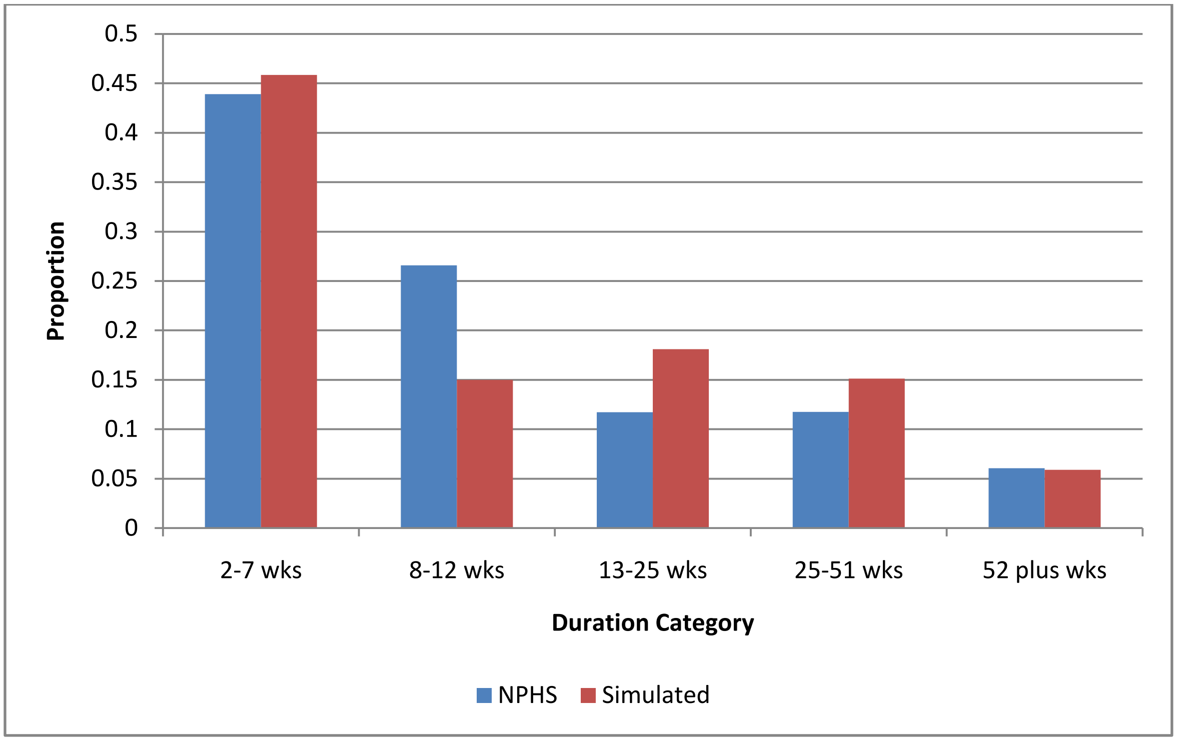

Figure 36. Age 66 or more, Pain, No Smoking and No Childhood Stressors

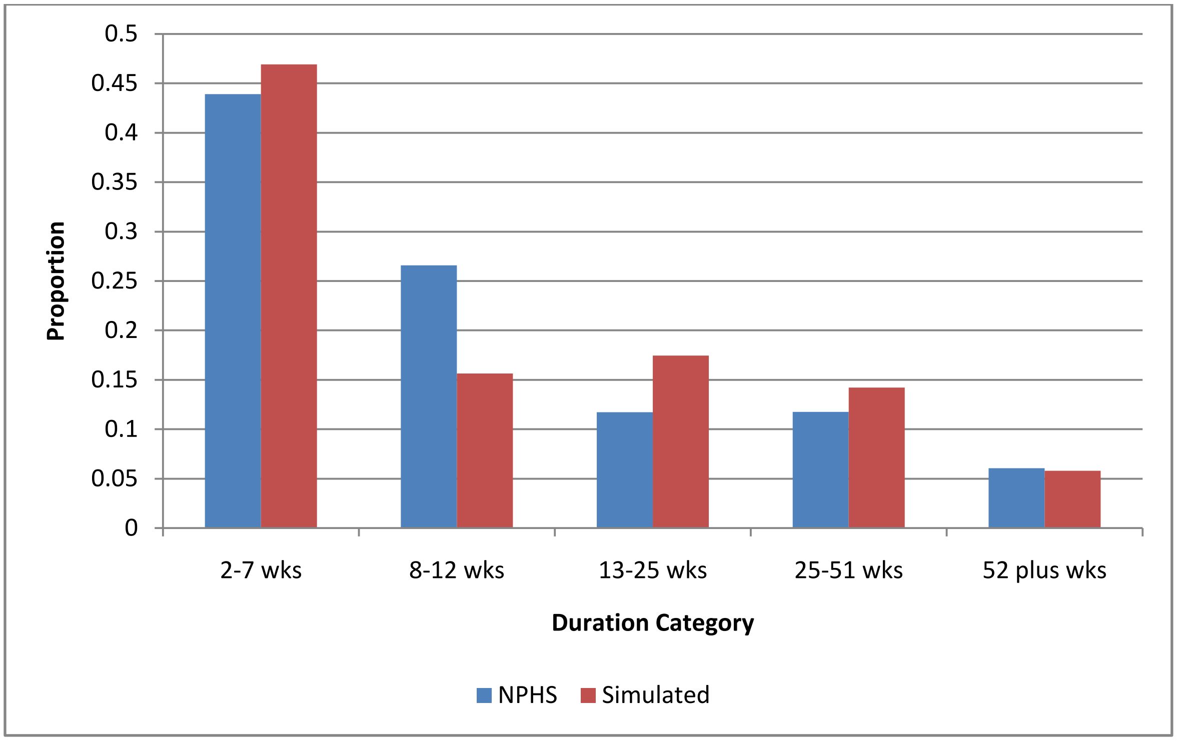

Figure 37. Age 66 or more, No Pain, Smoking and Childhood Stressors

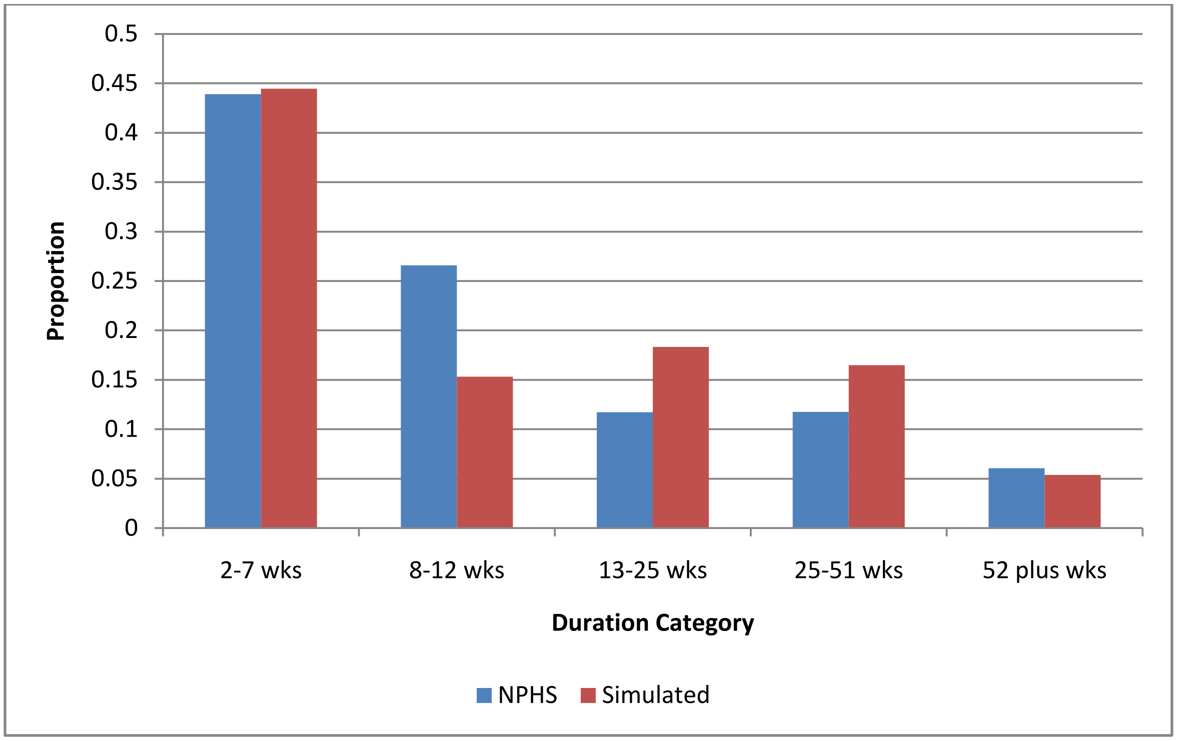

Figure 38. Age 66 or more, No Pain, Smoking and No Childhood Stressors

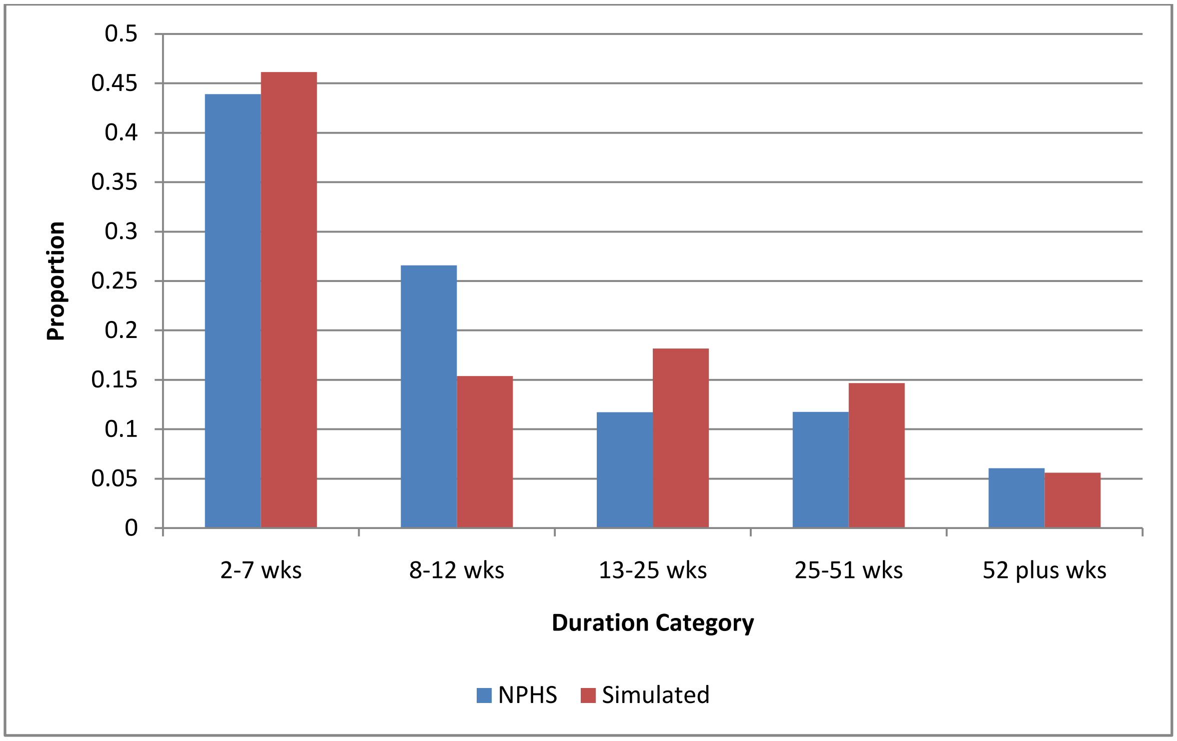

Figure 39. Age 66 or more, No Pain, No Smoking and Childhood Stressors

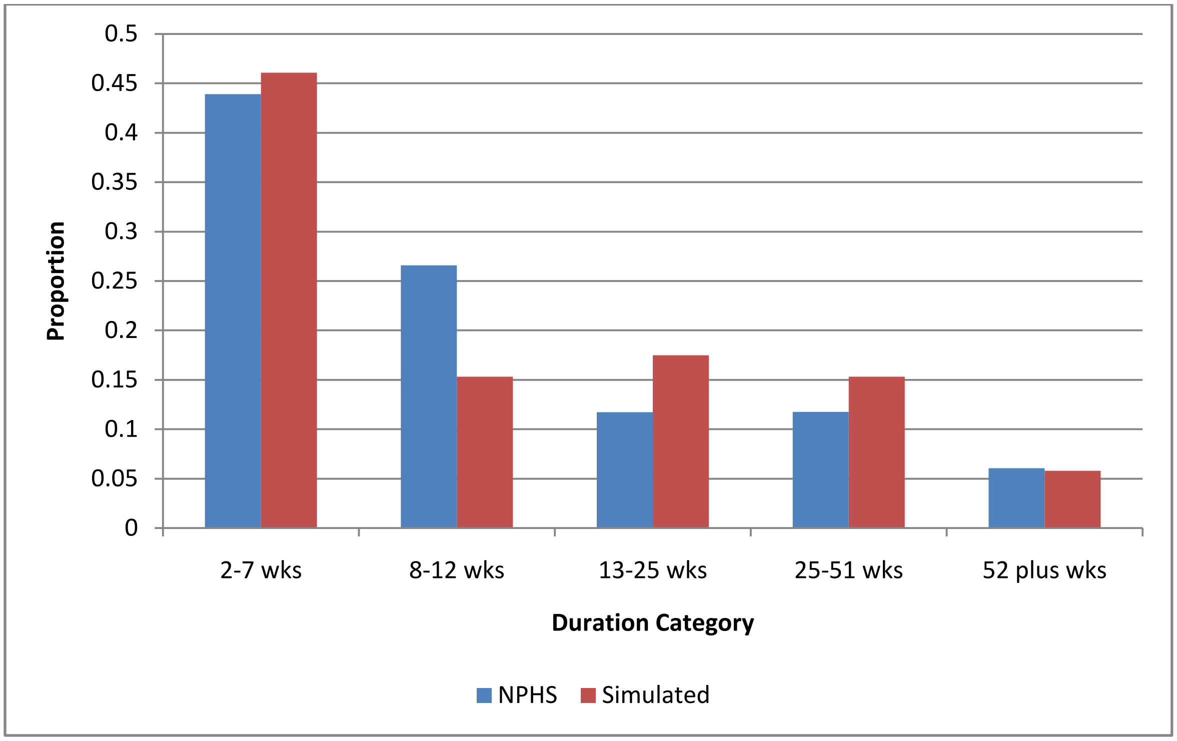

Figure 40. Age 66 or more, No Pain, No Smoking and No Childhood Stressors

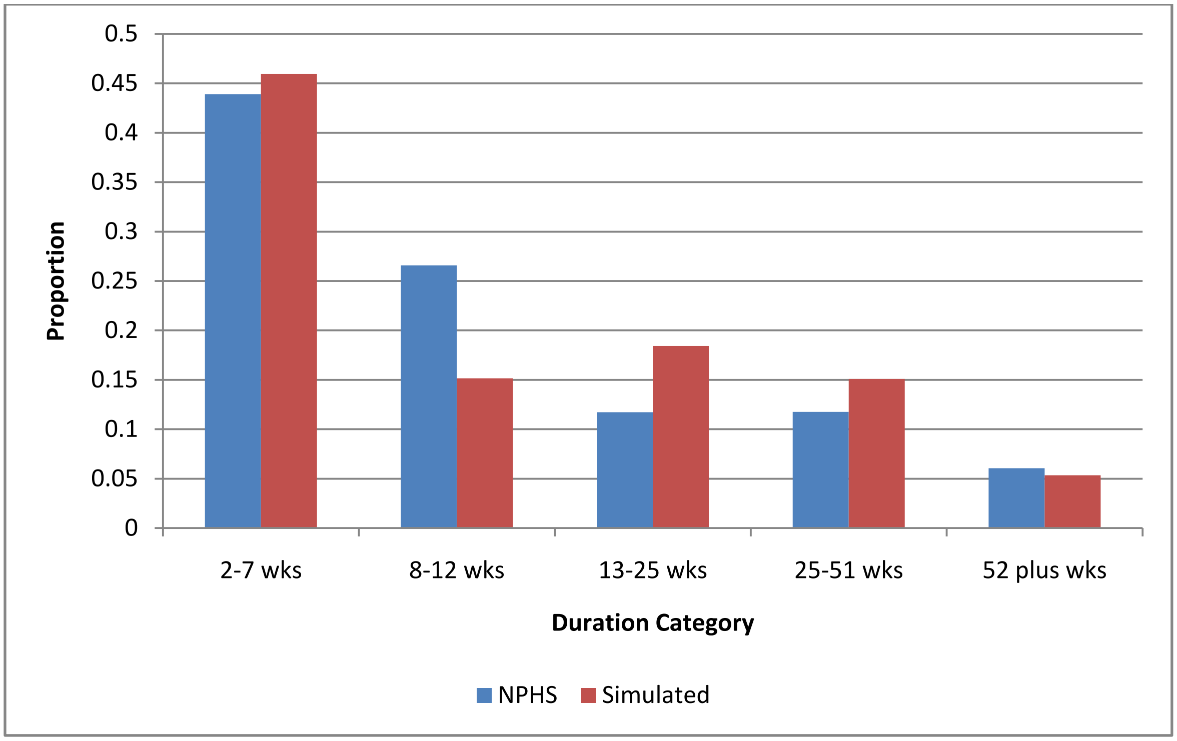

Supplement: Additional file 1 — Predicted episode durations. [file 1756-0500-3-231-S1.PDF]
